# A service evaluation of the uptake and effectiveness of a digital delivery of the NHS health check service

**DOI:** 10.1136/bmjopen-2024-091417

**Published:** 2024-11-09

**Authors:** Ruth Salway, Carlos Sillero-Rejon, Chloe Forte, Elisabeth Grey, Patricia Jessiman, Hugh McLeod, Rebecca Harkes, Paul Stokes, Frank De Vocht, Rona Campbell, Russell Jago

**Affiliations:** 1 Population Health Sciences, University of Bristol, Bristol, UK; 2 NIHR ARC West, Bristol, Bristol, UK; 3 Public Health, London Borough of Southwark, London, UK; 4 Prevention and Health Improvement, Cambridgeshire and Peterborough Joint Public Health Directorate, Cambridge, UK

**Keywords:** eHealth, Primary Health Care, Health economics, Electronic Health Records

## Abstract

**Abstract:**

**Objectives:**

To compare the uptake, effectiveness and costs of a digital version of the National Health Service (NHS) Health Check (DHC) to the standard face-to-face NHS Health Check (F2F).

**Participants and setting:**

A random sample of 9000 patients aged 40–74 eligible for an NHS Health Check in Southwark, England, between January and April 2023.

**Intervention and design:**

The DHC was an online tool with a health assessment section, an advice and support section, and a section on how to obtain and update follow-up physical measures (blood pressure, cholesterol, glycated haemoglobin (HbA1c)). 6000 patients from GP records were randomly allocated to receive a DHC invitation and 3000 to receive an F2F invitation. Those invited to DHC were able to choose F2F if they preferred.

**Outcomes:**

The primary outcome was the uptake of any type of health check, either a completed F2F appointment or completion of the DHC health assessment section, along with demographics and data on appointments, medications and referrals within the study period. QRISK3 and QDiabetes risk scores were calculated. Management and operation costs were estimated for F2F and DHC pathways.

**Results:**

Excluding participants who moved away or died, the DHC uptake to the health assessment section was 21% (1189/5705), with a further 3% (198/5705) choosing F2F, compared with 11% (305/2900) for F2F completion (p<0.001). The DHC uptake was lower among those from Black (14%) and Mixed (13%) compared with White (29%) ethnicities (p<0.001), and there was no evidence of higher DHC uptake among groups less likely to engage in NHS Health Checks. Of those who completed the health assessment, 60% (714) completed the support section, and 7% (84) completed the provision and updating of physical measures. Appointments, medications and referrals were lower among DHC service users than among F2F users (p<0.001). The estimated total management and operation costs for F2F were £154.80 per user, compared with total management and operation costs for DHC of £68.48 per user for health assessment only, £134.46 including the support section and £1479.01 per user with completed physical measures.

**Conclusions:**

The study suggests that a choice of Health Check pathways may potentially reduce pressures on the NHS. Cholesterol and HbA1c were not generally known, and the options to obtain and update these measures require further development for the DHC to be considered a viable comparable alternative to the F2F service for estimating cardiovascular disease and diabetes risk. Strategies are still needed to reach those groups not currently engaging with NHS Health Checks.

**Registration:**

This study was registered on the Open Science Framework: https://osf.io/y87zt.https://osf.io/y87zt

**Strengths and limitations of this study**

- The randomised design with a control group and large representative sample size provides a strong framework for inference.
- Our evaluation provides quantitative estimates of service uptake, risk identification and development, management and operation costs.
- Digital and face-to-face Health Checks used different invitation protocols, which reduces our ability to make direct comparisons on uptake.
- Data on further appointments, medications and referrals are for any purpose and may not be directly a result of a health check.
- Results from the supplementary 6 month user survey experienced low response rates and cannot be linked directly to the main data on demographic or health check outcomes.

## Introduction

 Cardiovascular disease (CVD) is a global public health priority,^1^ with a 2015 estimated cost of £9 billion in healthcare costs and £19 billion to society overall in England.^2^ In 2021, there were 136 616 deaths in England due to CVD, of which 33 481 were among those aged under 75.^3^ Similarly, around 4.2 million people in the UK were estimated to be living with type 2 diabetes in 2019, and the UK National Health Service (NHS) spends at least £10 billion per year on diabetes.^4^ Both CVD and type 2 diabetes are strongly associated with health inequalities,^5 6^ with those living in the most deprived areas almost four times more likely to die prematurely of CVD than someone in the least deprived, and health risk increases directly with the number of risk factors, including unhealthy behaviours.^7^ The Global Burden of Disease Study showed that 85% of CVD is preventable,^8^ with lifestyle changes such as increasing physical activity, stopping smoking, maintaining a healthy weight and low levels of alcohol consumption reducing the risk of CVD and type 2 diabetes. CVD burden is likely to be reduced more efficiently through preventive strategies such as screening for CVD risk factors, rather than curative strategies.^9 10^ Early treatment of these individuals at high risk of CVD may prevent the occurrence of life-threatening cardiovascular events and hospital admissions, resulting in potential health benefits and reduced CVD costs.^11^

The NHS has identified CVD prevention and management as a key priority in the NHS Long-Term Plan over the next ten years.^12^ An important part of this is improving the effectiveness of the NHS Health Check Programme,^13^ which aims to detect early signs of CVD and type 2 diabetes in adults aged 40–74 years. The Health Check assesses the top seven risk factors driving the burden of CVD and other non-communicable diseases: tobacco smoking, excess weight, physical inactivity, excess alcohol consumption, high blood pressure, high cholesterol and impaired glucose processing; and provides individuals with behavioural support and, where appropriate, treatment. The aim is to provide early identification and management of risk factors and to reduce health inequalities in CVD prevalence. Local authorities are required to offer all eligible individuals an NHS Health Check every 5 years, with specific tests and measures to be included in the risk assessment, and results communicated to the individual. The English Office for Health Improvement and Disparities target for Health Check uptake is 75% uptake. However, the national uptake of Health Checks has declined from 49% in 2013/14 to 39% in 2022/23 with large geographical variation.^14^ Those attending Health Checks include a higher proportion of women, those who are younger and those from the most affluent areas, although ethnic minority groups are also well represented.^15^ They are also more likely than non-attendees to be diagnosed with type 2 diabetes, hypertension and chronic kidney disease and to receive treatment such as statins and antihypertensives.^16^

The NHS is committed to digital transformation of health and social care, including specific aims for digital health checks and risk-based screening in England by 2028.^17^ However, evidence on the use of digital technology is limited. A 2018 rapid evidence synthesis of digital-first models of healthcare delivery found that uptake of digital alternatives to face-to-face (F2F) appointments was generally low, with users more likely to be younger, female and from higher income and educational backgrounds.^18^ However, it found that much of the existing data was qualitative, focusing on user perceptions or barriers to engagement,^19^ and there was a lack of evidence on quantitative outcomes. Thus, there is a need for empirical evidence on the benefits of digital solutions, especially around inequalities and cost-effectiveness.

This study is a service evaluation of a digital version of the NHS Health Check (DHC), developed by the London Borough of Southwark. Initial piloting of the DHC (which did not include a comparator group) found high engagement among those from more deprived areas and those who did not respond to a previous F2F health check offer. This evaluation compares the usual NHS Health Check procedure, a F2F Health Check, with the DHC, offered with a choice of F2F for those who prefer, in the form that would be implemented more widely if successful, as a complementary service to the existing programme. The aims of this study are as follows:

1. To first compare the uptake of DHC and F2F and second assess the extent to which the DHC is effective at engaging those groups that are under-represented in F2F.
2. To explore the extent to which the service overall is effective at encouraging people to take positive health actions.
3. To look at the effectiveness of the DHC in identifying those with increased risk of CVD and/or diabetes.
4. To estimate the costs associated with the DHC and F2F services.

## Methods

### Study design

The study protocol was pre-registered on the Open Science Framework,^20^ and this study forms part of the service evaluation of a digital NHS Health Check service in Southwark to compare the uptake of NHS health checks between those invited to DHC and those invited to F2F. The study population was all those eligible for an NHS Health Check in the northern half of the borough of Southwark, that is, aged 40–74 years; registered with a general practitioner (GP, a doctor who provides general health services to their local community through a GP surgery); have not had a Health Check in the previous 5 years; not registered as having coronary heart disease, chronic kidney disease, diabetes, hypertension, atrial fibrillation, transient ischaemic attack, familial hypercholesterolemia, heart failure, peripheral arterial disease or stroke; not currently receiving palliative care; not currently being prescribed statins for the purpose of lowering cholesterol; and not been found to have a 20% or higher risk of developing CVD over the next ten years (either in a previous Health Check or any other health service in England).^13^ A pragmatic random sample of 9000 eligible service users was drawn at a single point in time (January 2022) before the study began by the GP Federation, from the electronic patient health record system Education Management Information System (EMIS). The sample size reflected typical prevalence rates to ensure a reasonable-sized sample of those identified at high risk of CVD or diabetes and to allow analysis by subgroups. Of these, 3000 were randomly allocated to receive an invitation to F2F and 6000 to receive an invitation to the DHC, with the larger DHC sample to allow us to explore the DHC process in more detail, including movement through the pathway and in particular the uptake of physical measures. Invitations were issued in ten cohorts of 300 (F2F) and 600 (DHC) each between 24 January 2023 and 18 April 2023. Initial invitations were sent by SMS text message, followed by an SMS reminder 2 days later (DHC) or 1 week later (F2F). Those invited to DHC additionally received a letter and a final SMS reminder, 1 week and 2 weeks, respectively, after the initial invitation. The F2F invitation process corresponds to the standard procedure for F2F NHS Health Checks in the study area. This study used pseudonymised demographic and health data from patient records and was approved by the Integrated Research Application System, which provides permissions and approvals for health and social care/community care research in the UK. All those in the study sample were invited, with up to two reminders, to complete an online survey (see online supplemental file 2 for details of the questionnaire)6 months after their original Health Check invitation (ie, between July and September 2023). Respondents did not need to have completed a health check to fill in the follow-up survey. Completion was optional, and those taking part were offered entry to a prize draw to win one of ten £50 shopping vouchers. Respondents were presented with information on the purpose of the survey and how their data would be used and notified that submission of a survey would be taken as informed consent for the individual’s data to be used for research purposes. Data were routinely collected data and provided after allocation, so blinding was not needed, and adverse events were not recorded. There were no interim analyses and no changes to the protocol. This study received favourable opinions from the NHS Research Ethics Committee and Health Research Authority in January 2023 (Reference 22/EM/0280).

### Patient and public involvement

Eight residents aged between 40 and 59 years from Southwark’s Healthwatch network, a community group that champions the views of local users of health and social care services to influence how providers deliver care, were recruited to participate in a patient and public involvement group (PPI). The majority of the group was female and of Black ethnicity. The PPI group was set up when designing the research, and through online meetings, the group provided feedback and suggestions on the proposed study protocol and research materials. Two members of the group (one male, one female) then joined the project steering committee to continue giving advice from the perspective of a service user. The term ‘service user’ was chosen by our PPI representatives as the preferred language to refer to users of either pathway, and so we have used this term throughout this paper.

### Health check details

**Figure 1:**
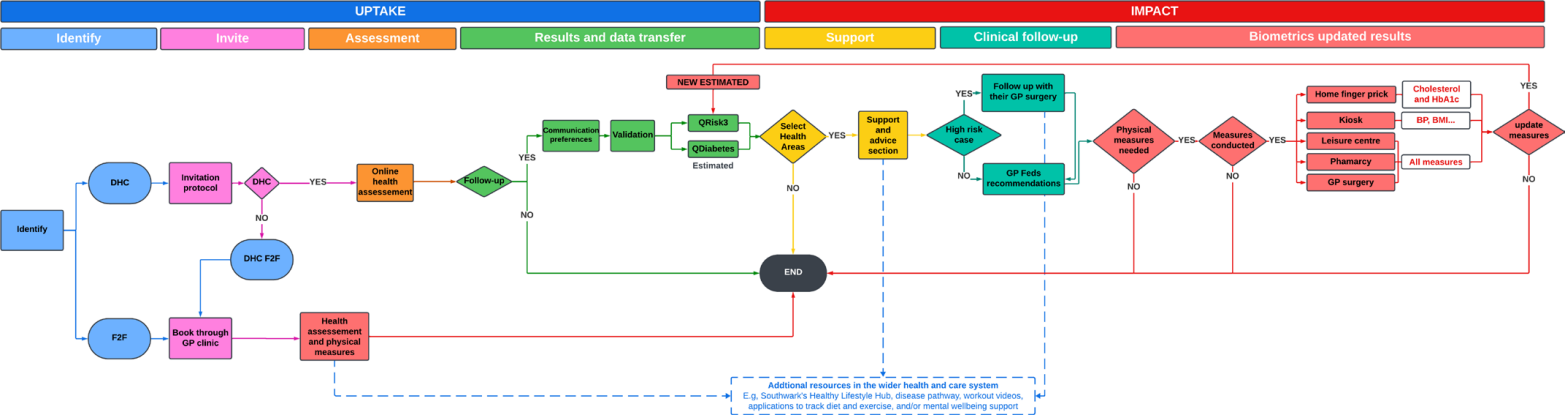
Overview of Face-to-Face and Digital Health Check pathways. DHC, Digital Health Check; F2F, face-to-face.

An overview of the F2F and DHC pathways is shown in figure 1. Those invited to F2F were asked to make an F2F appointment with a healthcare professional (HCP) at the GP surgery at which they are registered, via standard routes. During the F2F health check, the HCP conducts several physical measurements (height, weight, blood pressure, cholesterol and glycated haemoglobin (HbA1c) levels) and completes a structured template of standard health assessment questions on cardiovascular risk factors, incorporating the CVD QRISK3^21^ and QDiabetes^22^ screening questionnaires, smoking status, family history of coronary heart disease, alcohol consumption (alcohol use disorders identification test (AUDIT) score^23^) and physical activity (General Practice Physical Activity Questionnaire^24^). The QRISK3 and QDiabetes scores were calculated to estimate the 10 year risk of developing CVD and a diabetes risk assessment. Based on results, individuals may be offered further tests if necessary and/or behavioural advice and support for lifestyle changes to reduce risk.

Those invited to DHC were sent a unique encrypted link to the digital health check website in the invitation, where they were offered an F2F appointment instead of completing the DHC if they preferred. The digital tool consisted of a self-completed health assessment section, which followed the same structured template of questions as the F2F with the addition of the Whooley depression assessment,^25^ followed by an optional advice and support section. In the DHC health assessment section, service users were asked to enter physical measurements (height, weight, blood pressure, cholesterol, HbA1c) where known. Completion of the DHC health assessment section was considered to be the primary comparison point with a completed F2F health check. After completing this section, users were given personalised results based on their responses. These included their QRISK3 score (risk of developing a heart attack or stroke over the next 10 years) and QDiabetes score (risk of developing type-2 Diabetes over the next 10 years), estimated from the provided data or using age-sex population average values for measurements, which were not reported. The results also highlighted aspects of their lifestyle that could be improved and offered recommendations and links for further actions. Those service users whose results suggested they were at high risk were recommended to contact their GP for a non-urgent discussion of results and to access additional support; specifically those with excess alcohol consumption (AUDIT ≥8), underweight (body mass index (BMI) of <18.4), with obesity (BMI >30 for those of white ethnicity or >27.5 for Black or Asian ethnicities), high blood pressure (systolic 140–180 mmHg or diastolic 90–110 mmHg), high cholesterol (>7.5 mmol/L), high HbA1c (42–48 mmol/mol), high risk of developing a heart attack or stroke (QRISK3 >10) or high type 2 diabetes risk (QDiabetes >5.6). Those service users identified as at very high risk were advised to contact their GP clinic urgently, that is, those who were very underweight (BMI <16), with very high blood pressure (systolic >180 mmHg, diastolic >110 mmHg) or with very high HbA1c (>100 mmol/mol). Health check data were also sent to the GP Federation which updated patient records and followed up with individual GP surgeries where there were recommended actions.

After the health assessment section, DHC service users could continue to an optional advice and support section, where they were offered a list of health priorities based on their responses to the health assessment section: to find physical measures if these were not known, to stop smoking, to drink less alcohol, to achieve a healthy weight, to move more, to improve mental well-being and to improve blood pressure, cholesterol or blood sugar levels. Users were asked to select two health priorities on which to focus, given personalised advice and supporting information and services, depending on identified behavioural barriers and preferences. For example, a smoker might be directed to tools to manage cravings or a free phone consultation on smoking cessation services. Those service users who chose to find physical measures as one of their health priorities were given choices and directions to obtain measures via pharmacies, leisure centres, kiosks, GP surgeries or a home finger-prick blood test kit. Users who obtained physical measures were able to return to the DHC to update their responses and receive updated results.

### Data

Pseudonymised patient data on demographics (sex, age, ethnicity and England Index of Multiple Deprivation 2019 (IMD)^26^ quintile) and most recent health data (family history of CVD, smoking status, BMI, blood pressure, QRISK3 score and QDiabetes score) were extracted from EMIS for the full study sample. Age was grouped into 40–49 years, 50–59 years and 60+ years, and ethnicity was classified as White, Black, Asian, Mixed or Other. Deprivation was categorised as those from the most deprived areas (defined as residing in areas from the lowest IMD deprivation quintile) versus those from less deprived areas (not in the lowest quintile).

Data on completed F2F health checks between 24 January 2023 and 16 June 2023 were extracted for the sample, including the date of the health check and the QRISK3 score and QDiabetes scores. Pseudonymised DHC data on progress through different stages of the digital tool, cardiovascular risk factor data, QRISK3 and QDiabetes scores, health priorities chosen and recommendations given were transferred from the secure DHC service database and linked to EMIS demographic data via an anonymised ID. Data also recorded whether physical measures (height, weight, blood pressure, cholesterol and HbA1c) were provided at the initial time of the DHC, were updated later or were not provided at all. A completed health check was defined for F2F as attendance at a health check appointment and for DHC as completion of the digital health assessment section. Data on any GP appointments, medications and referrals within the study period until 16 June 2023 were extracted from EMIS. Note that due to limitations of data-sharing agreements, these relate to any appointments made within the time frame for any medical purpose and may not be directly as a result of a health check.

The user survey contained questions on respondent opinions of the health checks, motivation to attend health checks, satisfaction with services offered post-health check and any actions taken following health check completion(online supplemental file 2). No demographic data were collected, and survey responses were not linked to the main health check data. We estimated the costs associated with each pathway using resource and cost data supplied by the Southwark Council team who designed and implemented the DHC tool for the DHC pathway and data from the Southwark Council team responsible for the running of the standard F2F NHS Health Check programme and relevant literature for the F2F pathway.

### Analysis

All service users were classified according to their allocation and completion of the type of health check. Those invited to F2F were classified as ‘F2F completers’ if they attended a health check appointment before 16 June 2023 and ‘F2F non-completers’ otherwise. Those invited to complete DHC were classified as ‘DHC completers’ if they completed the digital health assessment section, as ‘DHC->F2F completers’ if they completed a F2F health check appointment before 16 June 2023 and as ‘DHC non-completers’ otherwise. A small minority (n=67) of those allocated DHC completed both F2F and DHCs; these were assigned to ‘completed DHC’ or ‘completed F2F’ depending on which occurred first. Progress through the DHC was summarised by the percentage of service users who reached each stage: clicking the link in the SMS to access the landing page, clicking the link on the landing page to start the digital health check tool, completing the health assessment section and completing the optional advice and support section (figure 1). As the analysis was primarily descriptive, missing data were handled by casewise deletion.

#### Completion rates and profiles of those completing DHC and F2F

Demographics of completer and non-completer profiles were summarised descriptively, with health summaries (smoking, BMI, blood pressure and risk of CVD or diabetes) for health check completers.

Completion (uptake) rates of any type of health check (irrespective of type allocated) were compared between those invited to F2F and DHC. Differences in the invitation process meant that those invited to DHC had a shorter gap between initial and second SMS and received a letter and final reminder SMS, so we also calculated DHC completion rates after 1 week (before the letter); this was equivalent to the timing of the second SMS for F2F. Completion of any heath check was compared between the two pathways by demographics (sex, age group, ethnicity and deprivation). To determine whether DHC engaged different groups to F2F, we compared completion rates by subgroups, by fitting separate logistic regression models with any type of health check completion as the outcome, main effects for allocated health check type and subgroup variable and an interaction term between allocated health check and subgroup to capture differences in subgroup patterns.

#### Positive health actions

The health priorities chosen in the DHC advice and support section were summarised descriptively and by whether the service user was at high risk with respect to weight, smoking, physical inactivity or alcohol. We also summarised data from the 6 month user survey by health check pathway, the types of advice reported by respondents and whether they reported taking any actions after completing the health check.

#### Completion of physical measures and identification of those at risk of CVD or diabetes

The reporting of physical measures (height, weight, cholesterol, blood sugar and blood pressure) from the DHC was summarised by whether service users knew their data at initial DHC completion, whether they updated it later or whether it was not known. We also compared those identified as at high risk of CVD or diabetes between the two pathways and fitted logistic regression models adjusted for age, sex, ethnicity, deprivation, BMI category and smoking status to determine if any differences were due to differences in health check uptake. From the 6 month user survey, we reported whether those survey participants who had completed DHC and were advised to obtain physical measures had ordered home blood test kits or attended a pharmacy, leisure centre or a blood pressure kiosk.

#### Follow-up with GP surgery

In the DHC, those identified at high or very high risk were advised to contact their GP surgery to make a non-urgent or urgent appointment for follow-up. We reported the percentage of those who were advised to make appointments, the percentage of those who choose following up with the GP as one of their health priorities and the percentage who attended an appointment at their GP surgery within the study time frame. Finally, we summarised those who made GP surgery appointments were prescribed medications or given referrals by health check pathway and by risk of CVD (medium/high CVD risk: QRISK3 >10) or diabetes (high diabetes risk: QDiabetes >5.6).

#### Cost analysis

Costs were categorised between development, management and operation.^27^ Development costs were those relating to the pre-study phases of DHC pathway evolution in Southwark (eg, DHC design and piloting). Management costs included the project management and service design, with estimated management costs allocated pro-rata across the 9000 service users in either of the DHC and F2F pathways. Operational costs were those associated directly with running the service and include identification and invitation to participate and delivery of the DHC and F2F pathways, including the cost of hosting and technical support for the digital service. The DHC pathway included both the digital service and obtaining and updating physical measures where these were not unknown. We allocated the DHC operational costs across three phases of the pathway, relating to completion of health assessment, digital support and obtaining and updating physical measures (see flow diagram and footnotes to table 2 and online supplemental table S13). The F2F health check unit cost was estimated using data reported by the Office for Health Improvement and Disparities.^28^

We estimated a potential scale-up scenario to illustrate how the study costs may change if delivered over a larger population. We used ONS population data^29^ to estimate the Southwark population of 107 000 aged 40–74 years and assumed that half would be invited to the DHC and F2F pathways over 4 years. We have assumed some economies of scale relating to study costs (see footnotes to table 2 and online supplemental table S12).

We combined activity and cost data to estimate the total cost for the DHC and F2F pathways, distinguishing between the three phases of the DHC pathway, relating to the completion of health assessment, digital support and obtaining and updating physical measures. The total cost per user was calculated as the estimated total cost divided by the number of service users who completed each stage of the health check pathway. The costs for those individuals who participated in both DHC and F2F pathways have been allocated to the pathway to which they were allocated. We estimated the cost of identifying high-risk users in both pathways, distinguishing between high-risk users identified from complete physical measurement data and ‘pseudo-high-risk’ users who were identified using population-based estimates of physical measures in calculating QRISK3 and QDiabetes scores where their data were incomplete. The total cost per high-risk individual was calculated as the estimated total pathway cost divided by the number of high-risk individuals identified. The estimated management costs were specific to this study setting and allocated pro-rata between DHC and F2F service users. Management costs have been omitted from other health check studies and would be comparatively low in standard F2F services.

## Results

Of the full sample, 395 participants were excluded by moving away or death (online supplemental figure S1). Of the remaining 8605, 31% were female, 50% were of white ethnicity and 26% were from the most deprived areas (online supplemental table S1). Around half (53%) were aged 40–49, a third (34%) aged 50–59 and the remainder (14%) were aged 60 or over. In total, 1692 (20%) patients completed a health check: 305 (11%) of those allocated to F2F completed a F2F health check, 198 (3%) allocated to DHC completed a F2F health check and 1189 (21%) allocated to DHC completed the DHC (online supplemental figure S1). The response rate for the 6 month user survey data was 8% with 308 (46%) respondents reporting completion of a health check (99 allocated to F2F, 99 allocated to DHC who chose F2F and 110 who completed DHC).

### Completion rates and profiles of those completing health checks

The profile of Health Checks completers included higher proportions of women, those from less deprived areas and those with a family history of CVD, compared with those who did not complete a Health Check (online supplemental table S2). DHC completers compared with F2F completers comprised fewer service users with overweight (35% compared with 40%) or obesity (17% vs 23%) and more men (DHC: 59%; F2F: 52%), from white ethnicity (DHC: 64%; F2F: 52%), and with low diabetes risk (DHC: 69%; F2F: 54%). Profiles of those directly invited to F2F health checks and those who chose F2F health checks after being invited to DHC were generally similar, but the latter included a higher percentage of those from the most deprived areas (DHC, chose F2F: 28%; F2F: 19%), those with obesity (DHC, chose F2F: 29%; F2F: 23%) or those at high risk of diabetes (DHC, chose F2F: 53%; F2F: 46%). Those who chose F2F rather than DHC included more users from ethnic minority groups and from the most deprived areas.

**Figure 2:**
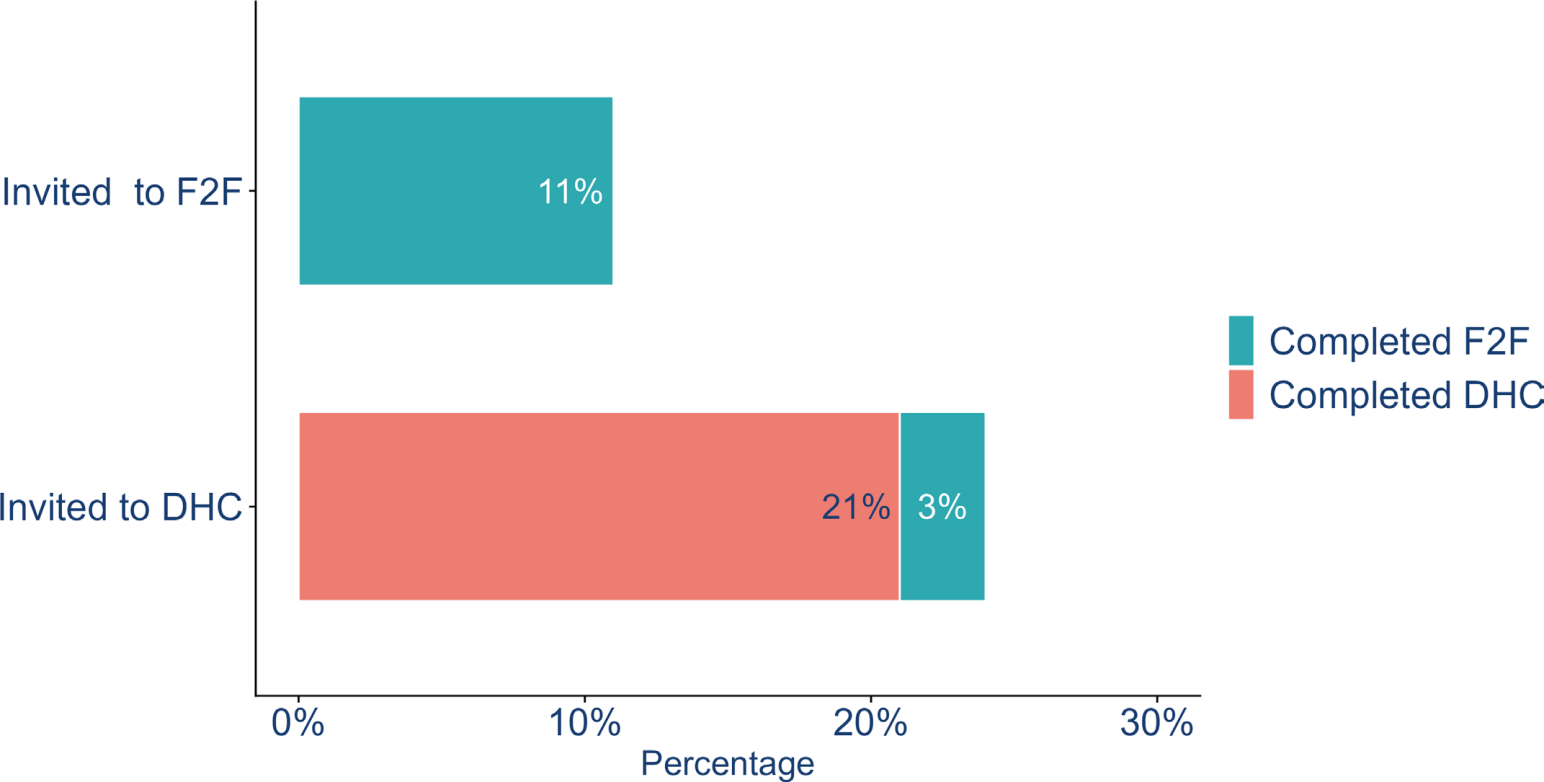
Uptake of face-to-face and Digital Health Check by type of health check allocated. F2F, face-to-face; DHC, Digital Health Check.

The completion rate was 11% for those allocated to F2F compared with 21% of those allocated to DHC who completed the DHC health assessment section (p<0.001), with a further 3% of these choosing to take up a F2F appointment instead (figure 2). Thus, 24% overall completed any health check after being invited to DHC. Note that differences in the invitation process mean that those invited to DHC received an extra letter and SMS; the DHC completion rate after 1 week (after the second SMS but before the letter) was 6% (online supplemental table S3).

For those allocated to F2F, completion among men was around half that among women at 8% and 16%, respectively (table 1). F2F completion was also lower among those from most deprived areas and those with no family history of CVD. Similar patterns for these variables were seen for those allocated to DHC, with lower completion among men, those from the most deprived areas and those with no family history of CVD. While all subgroup health check completion rates were higher for those allocated to DHC than for F2F, differences between subgroups were maintained across the Health Check pathways, with the exception of ethnicity (table 1). There were differences in completion of health checks by ethnicity for those allocated to DHC which were not present for those allocated to F2F (p<0.001), with health check completion among those allocated to DHC of 32% among white ethnicity but 19% and 17% for Black and Mixed ethnicities, respectively (allocated F2F: White 12%; Black: 12%; Mixed 9%). These completion rates refer to the completion of any health check within the allocated pathway; for comparison online supplemental table S4 shows completion rates for health check as allocated.

**Table 1:** Completion of any health check by allocated pathway by demographic and health risk subgroups

	Allocated to F2F		Allocated to DHC		F2F subgroup differences	Health check differences
	Total	% completed	Total	% completed	P value*	P value†
All	2900	11%	5705	24%		<0.001
Male	1967	8%	3939	20%		
Female	932	16%	1766	34%	<0.001	0.587
Age 40–49	1551	10%	2981	23%		
Age 50–59	949	11%	1943	25%		
Age 60+	400	11%	781	27%	0.741	0.916
White	1288	12%	2636	32%		
Black	553	12%	1094	19%		
Asian	258	12%	484	25%		
Mixed	324	9%	492	17%		
Other	214	7%	430	25%	0.198	<0.001
Less deprived‡	2144	12%	4236	26%		
Most deprived	753	8%	1465	19%	0.004	0.881
No CVD history	2011	8%	3928	20%		
Family CVD history	889	16%	1777	35%	<0.001	0.773

* p-value for whether there are subgroup differences for F2F.

† p-value for whether subgroup differences are the same for both F2F and DHC (health check interaction term).

‡ most deprived=lowest England Index of Multiple Deprivation (IMD) quintile.

CVD, cardiovascular disease

### Positive health actions

There was high engagement with the advice and support section of the DHC, with 60% of those completing the DHC also completing the support section. The most commonly chosen health priorities were to learn cholesterol and blood pressure (online supplemental table S5) with 42% and 35% of service users choosing these options, respectively. Other common choices were to achieve healthy weight (33%), move more (24%) and improve mental well-being (23%). Half or less of service users chose relevant health priorities when risk factors for weight, smoking, physical inactivity or alcohol consumption were above the threshold for intervention. Results from the 6 month survey showed similar priorities for participants who completed either DHC or F2F, with the most common options for F2F participants being to improve cholesterol (39%), followed by move more (22%) and achieve a healthy weight (12%; Online supplemental table S6). Around half of respondents reported taking action after the health check, with rates slightly higher for F2F, although sample sizes were small.

### Completion of physical measures and identification of those at risk of CVD or diabetes

The DHC resulted in low levels of data on physical measures, with 23% of service users reporting their blood pressure, 6% knowing cholesterol and 5% knowing HbA1c (online supplemental table S7). Finding out cholesterol and blood pressure were the top health priority choices (online supplemental table S5) with 57% selecting at least one option, but only 3% provided updated values to the DHC (online supplemental table S7). In the 6 month user survey, 92% of respondents who completed the DHC said they had been recommended to obtain their physical measures, and 53% of these reported completing a home blood test or visiting a pharmacy, leisure centre or blood pressure kiosk (online supplemental table S8), although note that estimates from the main DHC data and the 6 month user survey are not directly comparable.

For those completing F2F, 14% and 45% were identified as at risk of CVD and diabetes, respectively (online supplemental table S9). For those completing DHC, a similar proportion was identified to be at risk of CVD (17%), but fewer were identified as at high risk of diabetes (30%). Those completing DHC were less likely to be identified at risk of diabetes than F2F, and this difference remains when adjusting for demographics and other risk factors (online supplemental table S10). Only 7% of those completing DHC provided all the data required for the QRISK3 score (cholesterol and blood pressure measurements) or QDiabetes score (HbA1c glucose), with age-sex population averages used for measurements where these were not provided. While sample sizes are small, risk estimates for these service users are similar to those calculated overall.

### Follow-up with GP surgery

Of those completing DHC, 56% were identified as at high risk and recommended to make a non-urgent appointment with an HCP, and 1% were identified as at very high risk and advised to make an urgent appointment (online supplemental table S11). Of these, 55% and 90%, respectively, chose following up with their GP surgery as a health priority, and around half (53% and 50%) attended an appointment at their GP surgery within the study time frame. Note that numbers for urgent recommendations were very small, and so results should be treated with caution. Overall, those completing health checks via either pathway had higher further involvement with their GP surgery, in terms of appointments, medications and referrals, than those who did not (online supplemental table S12). Involvement was higher for F2F compared with DHC with appointments and referrals around twice as high (appointments: 88% for F2F compared with 47% for DHC and referrals 42% and 20%), and medications at 48% compared with 38% for DHC (p<0.001). Patterns were similar when comparing follow-ups for those identified as at high risk of CVD or diabetes, with higher levels of GP appointments and referrals for those receiving a F2F health check, although F2F estimates should be treated with caution due to small denominators.

### Cost analysis

The development costs associated with the DHC pathway, including previous piloting work before the current study, were estimated to total £261 000. This included website development and hosting, strategic services design, GP federation input, community biometric testing and clinical advice. The estimated study management costs totalled £80 720 (table 2). The operational costs of the DHC pathway were estimated to be £70 424 across the three phases of the pathway, relating to completion of health assessment (42%, £29,394), digital support (18%, £12,800) and obtaining and updating physical measures (40%, £28,230) (table 2 and online supplemental table S13). The operational costs of the F2F pathway were estimated to total £20 309. The estimated total costs per user completing each stage of the pathways are shown in table 2, and they reflect the level of engagement with users in the study. The estimated management and operation costs for completion of F2F appointments were £154.80 per user, compared with £68.48 per user for completion of the DHC health assessment section, £134.46 for health assessment and support sections and £1479.01 per user for the total DHC including updating physical measures.

**Table 2:** Estimated cost of the face-to-face and Digital Health Check for the study and a potential scale-up scenario

	Study				Potential scale-up scenario			
	N	Total cost (£)	Unit cost (£)	Total cost per user* (£)	N†	Total cost (£)	Unit cost (£)	Total cost per user* (£)
F2F								
Management‡	3000	26 907	8.97		53 500	26 750	0.50	
Uptake: identification and invitation§	3000	8810	2.94		53 500	77 040	1.44	
Health check assessment¶	305	11 499	37.70		11 000	414 700	37.70	
Total F2F: completed	305	47 215	49.61	154.80	11 000	518 490	39.64	47.14
DHC								
Management‡	6000	53 813	8.97		53 500	26 750	0.50	
Uptake: identification and invitation§	6000	15 094	2.61		53 500	73 515	1.44	
Digital assessment‡	1215	14 300	11.77		10 834	45 450	4.20	
Total DHC: assessment completed	1215	83 208	23.35	68.48	10 834	145 715	6.14	13.45
Lifestyle support accessed§	714	12 800	17.93		6367	44 110	6.93	
Total DHC: support section completed	714	96 008	41.28	134.46	6367	189 825	13.07	29.82
Physical measures recorded and updated§	84	28 230	336.07		749	74 722	99.76	
Total DHC: physical measures completed	84	124 237	377.35	1479.01	749	264 547	112.83	353.20

The unit cost data are the estimated costs per service user for each type of pathway expenditure. This is usually calculated by dividing the total estimated expenditure for each stage of the pathway by the number of service users engaged at that stage. The exception to this is the cost of the F2F health check assessment, which is an estimate derived from literature. The total unit costs at each completed stage of the pathway (grey cells) show the estimated cumulative cost per service user given the number of service users engaged at each stage of the pathway. These estimates provide insights into the costs of service provision, which complement the ‘total cost per user’ estimates and only account for the number of service users who completed each stage of the pathway.

* Study and scale-up: total cost per user is the estimated total cost divided by the number of service users who completed the stated stage of the pathway.

† Scale-up: 107 000 individuals 40–74 years, targeting 53 500 individuals in each pathway over 4 years.

‡ Study: local data allocated pro-rata between F2F and DHC users. Scale-up: Assumed unit cost of £0.50 per user.

§ Study: See Table S13online supplemental table S13 for cost sources and assumptions.

¶ Study and scale-up: unit cost of £37.70 for health check excluding invitation costs from Office for Health Improvement & Disparities^28^ .

Table 2 also shows estimated costs associated with a potential scale-up scenario in which half the Southwark target population is invited to the DHC and the remainder to the F2F pathway over a period of 4 years. We have assumed that the performance of the DHC and F2F pathways observed in this study remain the same when scaled up. The economies of the scale we have assumed reduce the managements costs for both pathways, and the operational costs in each of the three phases of the DHC pathway (table 2).

The DHC pathway identified 23 (<1% of those invited to DHC) high-risk service users with an estimated cost per high-risk service user identified of £5402 (online supplemental table S14). Additional 440 (7%) users were identified as pseudo-high-risk (ie, users for whom at least one of the physical measures was incomplete) in the DHC pathway. The estimated cost per user of identifying these pseudo-high-risk users was £189. The F2F pathway identified 128 (4%) high-risk service users and 18 (1%) pseudo-high-risk users. For the F2F pathway, the estimated cost of identifying high-risk and pseudo-high-risk individuals was the same (£323).

## Discussion

This service evaluation found that the uptake of the digital NHS health check in the London Borough of Southwark, as measured by the completion of the health assessment section, was twice the uptake among those allocated to the standard F2F NHS health check (21% vs 11%). Furthermore, 24% of those allocated to the DHC pathway completed a heath check of some type, with this rate with DHC consistently higher within subgroups. Moreover, the DHC costs to this point were estimated at 44% that of the completed F2F. However, while these results are promising and suggest that the DHC may have potential benefits, there are a number of issues that need to be explored and refined further before being assessed for wider implementation. Most notable is that while completion rates for the health assessment section were higher than F2F completion, the identification of high-risk users via the provision and updating of physical measures (blood pressure, cholesterol and HbA1c) was very low. As the primary aim of the NHS health check is early identification of those at risk of CVD and/or diabetes, the DHC in the form evaluated here does not collect the data required to provide high-quality assessments of risks. In addition, differences between the uptake in the DHC and F2F pathways may in part be due to differences in the invitation process, with over half of digital health checks completed after a final SMS reminder, which was not received by those invited to the F2F health check. There may, therefore, be benefits in optimising the invitation process, irrespective of the type of health check. Table 3 shows a list of key findings and the potential implications of these.

**Table 3:** Key findings and implications

Key finding	Potential implications
Differences in preferences for DHC and F2F pathways among those invited to DHC.	DHC should be considered as a complementary option rather than a replacement to the existing service and offer wider choice for users.
DHC does not reach those who do not engage with F2F health checks.	Strategies are still needed to reach those groups not currently engaging with NHS Health Checks.
Differences in the DHC uptake before and after final the SMS text reminder.	Optimisation of the invitation process may increase the uptake of NHS Health Checks regardless of the pathway chosen.
Poor provision and uptake of physical measures, from which key estimates of CVD and diabetes risk are calculated.	The process for obtaining physical measures requires further development before being assessed for wider use.DHC in its current form could be used to reduce pressure on the NHS and supplement the F2F pathway, for example, by providing pre-health check screening and extending the lifestyle change support offered.
Obtaining updated measures selected as a health priority by some but not all users.	Obtaining physical measures to calculate accurate estimates of CVD and diabetes risk should be a priority, and options should be offered to all users and not left to individual choice.
Despite the interest expressed in obtaining physical measures, very low levels of updated data resulted in very high cost per user.	The complex process from choosing how to obtain measures, the logistics of actually obtaining the measures (sending off for kits, finding kiosks, taking blood samples, returning samples) and then updating and integrating data into existing systems needs to be improved at all stages.
Health priorities indicated a strong demand for support for mental well-being.	Mental well-being is not currently part of the NHS Health Check, but could be considered for inclusion in future.
GP appointments and referrals were lower for DHC indicating poorer follow-up for those on the DHC pathway.	Systems need to ensure that DHC outcomes and any further actions required are automatically passed on to GP surgeries and are followed up by the GP surgery rather than relying on users.

CVDcardiovascular diseaseDHCDigital Health CheckGPgeneral practitionerNHSNational Health Service

One of the primary aims of this study was to assess the extent to which the DHC is effective at engaging those groups not been reached by the F2F Health Check. Previous research has found that those less likely to attend health checks are male, older and from more deprived areas,^15^ and we saw similar demographics in this study among both F2F and DHC non-completers. Of those invited to DHC, those choosing DHC rather than F2F were disproportionately male, white and from more affluent areas. These demographics are typical users of digital tools, and, with the exception of sex, of those who use digital alternatives compared with F2F appointments.^18^ We did not find any evidence of higher uptake of any type of health check among those groups less likely to engage in health checks. Moreover, we saw ethnicity differences in those allocated to DHC, which were not present in F2F, with lower health check uptake among those from Black and mixed ethnicities. This is particularly concerning as ethnic minority groups in the UK have been found to be at a higher risk both of CVD^30^ and CVD-related risk factors.^31^ However, the digital health check is not intended to replace the standard health check but provide a complementary service with patients being able to choose which form of Health Check to engage with. While overall it did not reach a different demographic overall, we did find demographic differences in the type of health check chosen among those invited to DHC. Approximately 15% of those invited to DHC chose to complete a standard health check at their GP surgery instead, with those choosing F2F more likely to be women, from ethnic minority groups and from the most deprived areas. Thus, a digital version of the health check is appealing to certain demographics, while F2F remains preferable to other groups. This suggests that it is important to retain both options to avoid digital health inequalities, especially as around half (52%) of GP surgeries in England have high levels of registered patients who do not tend to use digital tools and services.^32^ Offering a choice of pathway is likely to be important to reach the broadest range of population and potentially reduce pressures on the NHS, by allowing those who are younger and healthier to choose the DHC pathway, freeing up capacity and reducing waiting times for F2F health checks for those with higher need and/or risk. It is important to highlight that strategies to extend health checks to reach under-served groups are still needed.

The level of data on physical measures in the DHC pathway was very low, with over 90% of service users providing no data on cholesterol or HbA1c. While this in part reflects the limited development of this stage of the DHC, it also indicates a need to understand the low levels of engagement and find ways to improve uptake of this stage. Consequently, the CVD and diabetes risk assessments for the majority of users are based on population average measures and may not reflect their true risks. While the DHC tool does highlight where results are based on estimated data, there may be some service users who are falsely reassured or worried by these results. In particular, DHC service users are much less likely to be identified as at risk of diabetes compared with F2F users, even after adjusting for demographics and other risk factors. This suggests that there may be a substantial minority of DHC users who have received an inaccurate assessment of low risk of diabetes. While those service users who did not know their physical measures were offered options to find out and update their results, the onus was on the user to both obtain measurements and update records and very few did so. The top health priorities selected were to obtain cholesterol and blood pressure measures indicating a desire for DHC service users to find out their measures, and survey data suggest that more people may have obtained or started to obtain follow-up measurements than updated the results. However, the low levels of updated measures indicate that there were issues with this process. Users who did not know their physical measures were offered the option to obtain them as a health priority, but they were only given details of how to do so if they selected this as one of their health priorities. Moreover, the user survey suggests there may be a substantial number of service users who obtained but did not update measurements in the DHC. Without updating results, neither users nor their GP surgeries have access to updated data, including updated estimates of CVD and diabetes risks. Furthermore, due to the limited uptake, the overall costs involved in providing options and processing data for obtaining updated physical measures were very high per high-risk user identified, and highlight a need for further work on the physical measurements phase of the DHC, motivated by the observed engagement with the DHC. This includes reviewing the popularity and cost of different options offered, encouraging uptake, exploring the process between intentions and actions and ensuring that resulting measures are properly uploaded and integrated into GP records. For example, better automation and linking of systems could help remove the reliance on the user to actively facilitate multiple parts of this process would be beneficial, especially for those service users who may find it harder to arrange or prioritise these. The NHS App provides patients with access to their health records, and as its use is becoming more widespread, this may result in more users being able to provide accurate physical measures from their records and potentially offer a centralised platform for integrating the DHC into standard pathways. Further investment in blood tests for cholesterol and HbA1c may also increase the uptake of home testing options. Once these processes have been further developed and piloted, it is likely that the cost per high-risk user identified for this part of the DHC pathway will reduce. In addition, there may be further downstream benefits not included in this analysis, for example, due to preventing unplanned hospital admissions from an acute cardiac event. However, until then, an alternative approach may be to focus on using the more successful phases of the DHC to supplement the F2F route, for example, to provide a low-cost pre-screening for low-risk individuals, as well as extended further support for behavioural change after a completed health check. Although we estimated costs associated with a potential scale-up scenario for the DHC and F2F pathways, the need to improve the physical measures section of the DHC pathway before any further rollout is such that the estimated performance and costs of the DHC would necessarily change.

One area where the DHC performed well was the optional advice and support section, with over 60% of those who completed the health assessment section continuing to this phase. This phase of the online tool is designed to mirror and extend the advice and signposting to further information part of the F2F health check, which is recommended by the NHS Health Check Best Practice Guidance.^13^ However, it is not clear that this always takes place, with a pre-COVID-19 national study reporting that only 16% of health check attendees were coded as having received general lifestyle and behavioural advice,^15^ although they report high levels of missing data and this will likely vary depending on how the F2F programme is delivered within a local authority; note that our survey of local users reported much higher levels of advice received for F2F participants (around 80%). However, even when advice is offered, current guidance recommends the delivery of only ‘very brief advice’ to ‘extended brief intervention’, depending on an individual need.^13 33^ Thus, the DHC offers a clear opportunity to provide more detailed support and direct links to support services for all, without staff-related time limits. Users were able to choose two or more health priorities to focus on, with the most common choices after obtaining physical measures to achieve a healthy weight and move more. There was a high demand for advice around mental well-being, which is not currently assessed in the F2F pathway. However, user choices were not always targeted on the most effective lifestyle changes; for example, less than half of smokers prioritised stopping smoking, despite smoking being the leading preventable cause of illness and premature death in the UK.^34^ In addition, for effective identification of those at risk of CVD or diabetes, obtaining physical measures should be a priority for all service users, but less than half chose this as a health priority. For those who did choose physical measures as a priority, interest focused on cholesterol and blood pressure, rather than blood sugar, although we found that DHC service users were much less likely to be identified as at risk of diabetes. In future, regardless of how this is implemented, obtaining physical measures should be prioritised for all users and not left to individual choice. Despite the high engagement with the advice and support section itself, there was no evidence from the user survey that it led to more action taken than those on the F2F pathway, although the generalisability of the user survey is limited. Furthermore, GP appointments and referrals, and to a lesser degree medications, were all less frequent in the DHC compared with F2F pathway, including among those identified at higher risk. This may be due to the ease of arranging further appointments while at the GP surgery during or after a F2F health check. It is also possible that those who prefer remote interactions may be more likely to choose DHC over F2F and also less likely to follow-up with a F2F appointment, which may contribute. Thus, for DHC service users, it is important that systems are in place to ensure that GP surgeries are notified of any required follow-ups and that these are arranged in a timely fashion, without requiring users to chase these.

This study has a number of limitations. As we have described, there were differences in the two pathways, in particular as those allocated to DHC had the option of choosing F2F, while those allocated to F2F were not able to opt for DHC. This may introduce sampling bias and thus limits our ability to make a direct comparison between F2F and DHC. Differences in the invitation process may also have contributed to the differences in uptake, with descriptive analysis suggesting that the additional SMS reminder may have boosted uptake. Unfortunately, we do not have data to explore this further. Due to data-sharing agreements, we were also unable to attribute GP appointments, referrals and medications as a direct result of health checks. However, this limitation applies equally to both F2F and DHC pathways, and as the provided levels of unrelated appointments are similar, the difference will be unbiased. It is also possible that those who prefer remote interactions may be more likely to choose DHC over F2F and also less likely to follow-up with a F2F appointment. In addition, results from the supplementary 6 month user survey experienced low response rates and cannot be linked directly to the main data on demographic or health check outcomes. We also have limited data on the barriers to obtaining physical measures. Finally, we recognise that there are more advanced digital health interventions being developed, which may have further benefits, but this evaluation focuses specifically on a relatively simple online tool with self-assessment forms of the type advocated in UK health policy.^17^ While we include a SPIRIT checklist (online supplemental file 3), some information is not available as this was a service evaluation and not a clinical trial.

## Conclusion

The study suggests that a choice of Health Check pathways may potentially reduce pressures on the NHS. Cholesterol and HbA1c were not generally known, and the options to obtain these measures require further development for the DHC to be considered a viable comparable alternative to the F2F service for estimating CVD and diabetes risk. Strategies are still needed to reach those groups not currently engaging with NHS Health Checks.

## supplementary material

10.1136/bmjopen-2024-091417 online supplemental file 1

10.1136/bmjopen-2024-091417 online supplemental file 2

10.1136/bmjopen-2024-091417 online supplemental file 3

## Data Availability

Data are available upon reasonable request.

## References

[1] GBD Causes of Death Collaborators (2018). Global, regional, and national age-sex-specific mortality for 282 causes of death in 195 countries and territories, 1980-2017: a systematic analysis for the Global Burden of Disease Study 2017. Lancet.

[2] Wilkins E (2017). European Cardiovascular Disease Statistics.

[3] British Heart Foundation (2023). Analysis of official uk mortality data in heart and circulatory disease statistics.

[4] Whicher CA, O’Neill S, Holt RIG (2020). Diabetes in the UK: 2019. Diabet Med.

[5] Public Health England (2019). Health Matters: Preventing Cardiovascular Disease.

[6] Fletcher B, Gulanick M, Lamendola C (2002). Risk factors for type 2 diabetes mellitus. J Cardiovasc Nurs.

[7] Khaw K-T, Wareham N, Bingham S (2008). Combined impact of health behaviours and mortality in men and women: the EPIC-Norfolk prospective population study. PLoS Med.

[8] Institute for Health Metrics and Evaluation (IHME) (2018). Findings from the Global Burden of Disease Study 2017.

[9] Kahn R, Robertson RM, Smith R (2008). The impact of prevention on reducing the burden of cardiovascular disease. Diabetes Care.

[10] Labarthe D (2011). Epidemiology and Prevention of Cardiovascular Diseases: A Global Challenge.

[11] Oude Wolcherink MJ, Behr CM, Pouwels XGLV (2023). Health Economic Research Assessing the Value of Early Detection of Cardiovascular Disease: A Systematic Review. Pharmacoeconomics.

[12] National Health Service (NHS) (2019). NHS Long Term Plan.

[13] Public Health England (2019). NHS Health Check Best Practice Guidance: For Commissioners and Providers.

[14] NHS Fingertips (2022). NHS health check data profile. https://fingertips.phe.org.uk/profile/nhs-health-check-detailed/data#page/1.

[15] Patel R, Barnard S, Thompson K (2020). Evaluation of the uptake and delivery of the NHS Health Check programme in England, using primary care data from 9.5 million people: a cross-sectional study. BMJ Open.

[16] Robson J, Garriga C, Coupland C (2021). NHS Health Checks: an observational study of equity and outcomes 2009-2017. Br J Gen Pract.

[17] Department of Health & Social Care (2022). Policy Paper: A Plan for Digital Health and Social Care.

[18] Rodgers M, Raine G, Thomas S (2019). Informing NHS policy in ‘digital-first primary care’: a rapid evidence synthesis. Health Serv Deliv Res.

[19] O’Connor S, Hanlon P, O’Donnell CA (2016). Understanding factors affecting patient and public engagement and recruitment to digital health interventions: a systematic review of qualitative studies. BMC Med Inform Decis Mak.

[20] Jago R Evaluation of the southwark nhs health check service.

[21] Hippisley-Cox J, Coupland C, Brindle P (2017). Development and validation of QRISK3 risk prediction algorithms to estimate future risk of cardiovascular disease: prospective cohort study. BMJ.

[22] Hippisley-Cox J, Coupland C (2017). Development and validation of QDiabetes-2018 risk prediction algorithm to estimate future risk of type 2 diabetes: cohort study. BMJ.

[23] Saunders JB, Aasland OG, Babor TF (1993). Development of the Alcohol Use Disorders Identification Test (AUDIT): WHO Collaborative Project on Early Detection of Persons with Harmful Alcohol Consumption--II. Addiction.

[24] Department of Health (2009). The general practice physical activity questionnaire: a screening tool to assess adult physical activity levels, within primary care.

[25] Whooley MA, Avins AL, Miranda J (1997). Case-finding instruments for depression. Two questions are as good as many. J Gen Intern Med.

[26] Ministry of Housing Communities and Local Government (2019). English indices of deprivation 2019. https://www.gov.uk/government/statistics/english-indices-of-deprivation-2019.

[27] Severens J, Wensing M, Grol R, Grimshaw J (2020). Improving Patient Care: The Implementation of Change in Health Care.

[28] Office for Health Improvement & Disparities (2021). Review of the nhs health check.

[29] Office for National Statistics UK (2023). https://www.ons.gov.uk/peoplepopulationandcommunity/populationandmigration/populationestimates.

[30] Lip GYH, Barnett AH, Bradbury A (2007). Ethnicity and cardiovascular disease prevention in the United Kingdom: a practical approach to management. J Hum Hypertens.

[31] Ho FK, Gray SR, Welsh P (2022). Ethnic differences in cardiovascular risk: examining differential exposure and susceptibility to risk factors. BMC Med.

[32] Citizens Online (2020). Age and digital exclusion risk: a map of gp surgeries in england. https://www.citizensonline.org.uk/digital-inclusion/digital-inclusion-digital-uptake-gps.

[33] National Institute for Health and Care Excellence (2014). Behaviour change: individual approaches (PH49).

[34] Allender S, Balakrishnan R, Scarborough P (2009). The burden of smoking-related ill health in the UK. Tob Control.

