# Supplementary material for: A service evaluation of the uptake and effectiveness of a digital delivery of the NHS health check service
Source: BMJ Open. 2024 Nov 9;14(11):e091417. doi: 10.1136/bmjopen-2024-091417 (PMC11552007; doi:10.1136/bmjopen-2024-091417)
Supplement: online supplemental file 1 [file bmjopen-14-11-s001.docx]

# An evaluation of the uptake and effectiveness of a digital delivery of the NHS Health Check service: a randomised controlled study

# Supplementary Tables

Ruth Salway, Carlos Sillero-Rejon, Chloe Forte, Lis Grey, Tricia Jessiman, Hugh McLeod, Rebecca Harkes, Frank de Vocht, Rona Campbell, Russell Jago

**^*^ Corresponding author**: Ruth Salway ([ruth.salway@bristol.ac.uk](mailto:ruth.salway@bristol.ac.uk))

# List of Figures

**Figure S1** Participant flow diagram

# List of Tables

**Table S1:** Demographics and missing data from GP records

**Table S2:** Profiles of health check completers and non-completers

**Table S3:** Uptake of Face-to-Face (F2F) and Digital Health Check (DHC) by initial invitation

**Table S4:** Uptake of Face-to-Face (F2F) and Digital Health Check (DHC) by demographic and health risk subgroups

**Table S5:** Percentage of those completing digital health check (DHC) who chose each option as one of their health priorities

**Table S6:** Advice offered and whether any action taken for survey participants in each type of health check (results from six month survey)

**Table S7:** Completion of physical measurement data in digital health check (DHC) : N = 1189 DHC completers

**Table S8:** Completion of physical measurements in digital health check among survey participants (results from six month survey)

**Table S9:** Service users identified as high risk by health check type

**Table S10:** Odd ratios of CVD and diabetes high risk for DHC versus F2F, adjusted for demographics

**Table S11:** Recommended GP follow-up and outcomes for Digital Health Check (DHC)

**Table S12:** GP Follow-ups by health check type completed

**Table S13:** F2F and DHC estimated cost data used in Table 2

**Table S14:** Costs associated with identifying those at high-risk by pathway

**Figure S1** Participant flow diagram

**
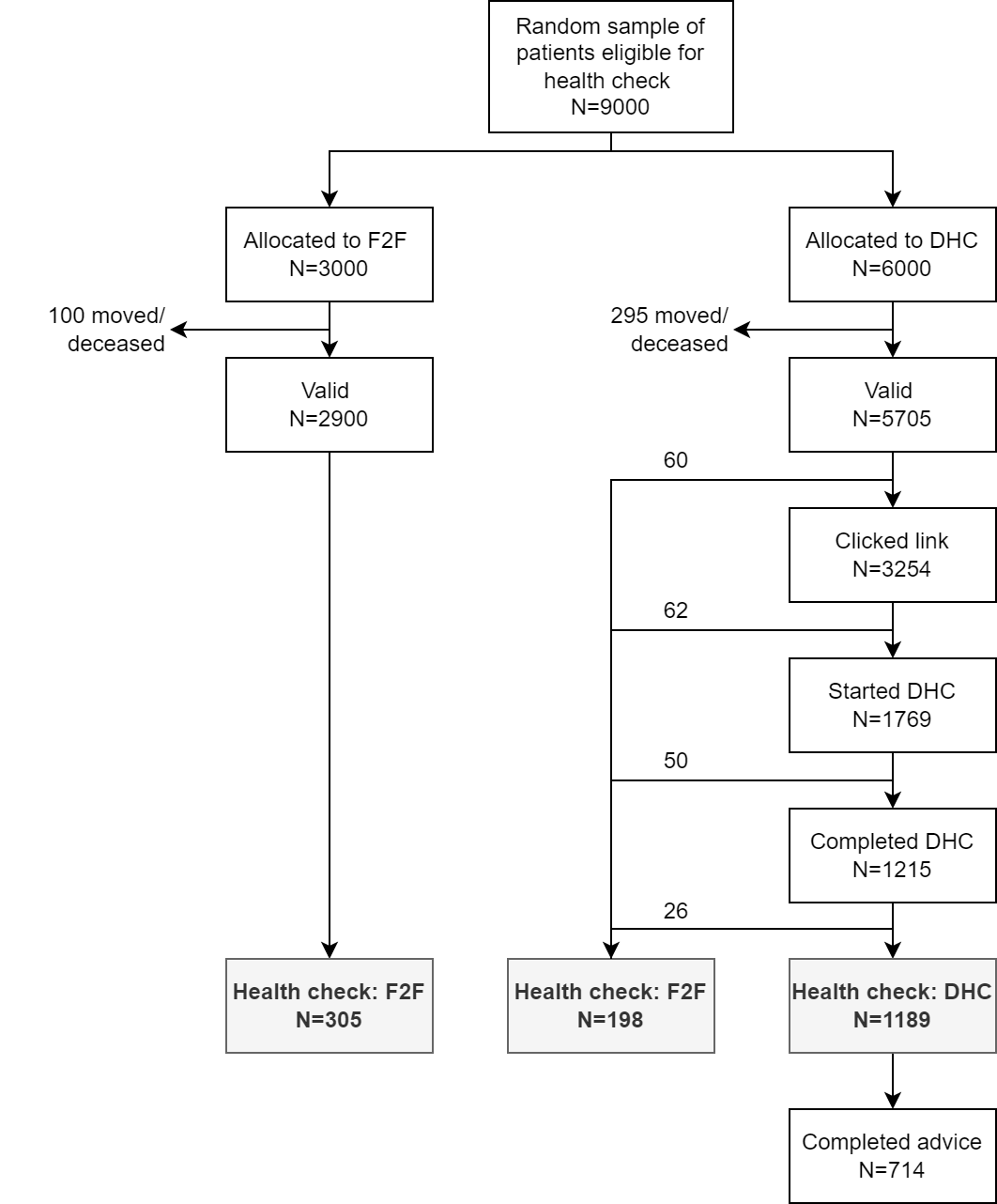
**

**Table S1: Demographics and missing data from GP records**

|  |  | Allocated F2F | | | | Allocated DHC | | | |
| --- | --- | --- | --- | --- | --- | --- | --- | --- | --- |
|  |  | Total | | Missing | | Total | | Missing | |
|  |  | N | % | N | % | N | % | N | % |
| **Gender** | |  |  | 1 | <1% |  |  | 0 | 0% |
|  | Male | 1967 | 68% |  |  | 3939 | 69% |  |  |
|  | Female | 932 | 32% |  |  | 1766 | 31% |  |  |
| **Age** | |  |  | 0 | 0% |  |  | 0 | 0% |
|  | Aged 40-49 | 1551 | 53% |  |  | 2981 | 52% |  |  |
|  | Aged 50-59 | 949 | 33% |  |  | 1943 | 34% |  |  |
|  | Aged 60+ | 400 | 14% |  |  | 781 | 14% |  |  |
| **Ethnicity** | |  |  | 263 | 9% |  |  | 569 | 10% |
|  | White | 1288 | 49% |  |  | 2636 | 51% |  |  |
|  | Black | 553 | 21% |  |  | 1094 | 21% |  |  |
|  | Asian | 258 | 10% |  |  | 484 | 9% |  |  |
|  | Mixed | 324 | 12% |  |  | 492 | 10% |  |  |
|  | Other | 214 | 8% |  |  | 430 | 8% |  |  |
| **Deprivation (IMD)** | |  |  | 3 | <1% |  |  | 4 | <1% |
|  | Less deprived | 2144 | 74% |  |  | 4236 | 75% |  |  |
|  | Most deprived | 753 | 26% |  |  | 1465 | 26% |  |  |
| **Family CVD history** | | | | 0 | 0% |  |  | 0 | 0% |
|  | No | 2011 | 69% |  |  | 3928 | 69% |  |  |
|  | Yes | 889 | 31% |  |  | 1777 | 31% |  |  |

F2F = Face-to-face health check; DHC = digital health check; IMD = index of multiple deprivation; CVD = cardiovascular disease; BMI = body mass index

**Table S2: Profiles of health check completers and non-completers**

|  | F2F | | DHC |  |  |  |
| --- | --- | --- | --- | --- | --- | --- |
|  | F2F Non-completers | | F2F Completers | DHC Non-completers | DHC -> F2F completers | DHC Completers |
| N | 2595 | | 305 | 4318 | 198 | 1189 |
| % female | 30% | | 48% | 27% | 53% | 41% |
| % aged 40-49 | 54% | | 51% | 53% | 45% | 51% |
| % aged 50-59 | 33% | | 34% | 34% | 41% | 34% |
| % aged 60+ | 14% | | 15% | 13% | 14% | 15% |
| % white | 48% | | 52% | 47% | 48% | 64% |
| % lowest IMD quintile | 27% | | 19% | 28% | 28% | 19% |
| % family CVD history | 29% | | 48% | 27% | 48% | 44% |
| % current smoker |  | | 15% |  | 15% | 16% |
| % healthy weight |  | | 37% |  | 37% | 48% |
| % overweight |  | | 40% |  | 34% | 35% |
| % obese |  | | 23% |  | 29% | 17% |
| % high blood pressure |  | | 15% |  | 17% | 14% |
| % high QRISK3 score |  | | 15% |  | 18% | 15% |
| % high Qdiabetes score | |  | 46% |  | 53% | 31% |

F2F = Face-to-face health check; DHC = digital health check; IMD = index of multiple deprivation; CVD = cardiovascular disease

Note: health risk factors are taken from health checks, and so are not available for non-completers.

**Table S3: Uptake of Face-to-Face (F2F) and Digital Health Check (DHC) by initial invitation**

|  |  | All | | Completed health check before letter/final SMS | |
| --- | --- | --- | --- | --- | --- |
|  |  | N | % | N | % |
| F2F | Total | 2900 |  |  |  |
|  | Completed F2F | 305 | 11% |  |  |
| DHC | Total | 5705 |  | 5705 |  |
|  | Completed DHC | 1148 | 21% | 360 | 6% |
|  | Completed F2F | 172 | 3% |  |  |
|  | Completed both | 67 | 1% |  |  |

SMS = text message

**Table S4: Uptake of** **Face-to-Face (F2F) and Digital Health Check (DHC) by demographic and health risk subgroups**

|  | Completed F2F | | Completed DHC | | F2F subgroup differences | Health check differences |
| --- | --- | --- | --- | --- | --- | --- |
|  | Total | % completed | Total | % completed | p-value^1^ | p-value^2^ |
| All | 2900 | 11% | 5705 | 21% |  | <0.001 |
| Male | 1967 | 8% | 3939 | 18% |  |  |
| Female | 932 | 16% | 1766 | 28% | <0.001 | 0.160 |
| Age 40-49 | 1551 | 10% | 2981 | 20% |  |  |
| Age 50-59 | 949 | 11% | 1943 | 21% |  |  |
| Age 60+ | 400 | 11% | 781 | 24% | 0.741 | 0.838 |
| White | 1288 | 12% | 2636 | 29% |  |  |
| Black | 553 | 12% | 1094 | 14% |  |  |
| Asian | 258 | 12% | 484 | 22% |  |  |
| Mixed | 324 | 9% | 492 | 13% |  |  |
| Other | 214 | 7% | 430 | 23% | 0.198 | <0.001 |
| Less deprived^3^ | 2144 | 12% | 4236 | 23% |  |  |
| Most deprived | 753 | 8% | 1465 | 15% | 0.004 | 0.707 |
| No CVD history | 2011 | 8% | 3928 | 17% |  |  |
| Family CVD history | 889 | 16% | 1777 | 29% | <0.001 | 0.416 |

CVD = cardiovascular disease

^1^ p-value for whether there are subgroup differences for F2F

^2^ p-value for whether subgroup differences are the same for both F2F and DHC (health check interaction term)

^3^ most deprived= lowest England Index of Multiple Deprivation (IMD) quintile

**Table S5: Percentage of those completing digital health check (DHC) who chose each option as one of their health priorities**

|  | N | % of those completing advice section | % of those at increased risk^1^ |
| --- | --- | --- | --- |
| Learn cholesterol | 372 | 42% |  |
| Learn blood pressure | 309 | 35% |  |
| Achieve healthy weight | 295 | 33% | 56% |
| Move more | 213 | 24% | 37% |
| Mental well-being | 203 | 23% |  |
| Learn blood sugar | 106 | 12% |  |
| Improve blood pressure | 76 | 9% | 48% |
| Drink less alcohol | 75 | 9% | 34% |
| Stop smoking | 57 | 6% | 44% |
| Improve cholesterol | 39 | 4% | 63% |
| Improve blood sugar | 17 | 2% |  |

^1^ denominator is those at increased risk with respect to this priority e.g. % of smokers who chose ’stop smoking’ or % of those with obesity who chose ‘achieve healthy weight’.

**Table S6: Advice offered and whether any action taken for survey participants in each type of health check (results from six month survey)**

|  | F2F  (N=99) | | DHC chose F2F  (N=99) | | DHC  (N=110) | |
| --- | --- | --- | --- | --- | --- | --- |
|  | N | % | N | % | N | % |
| Learn cholesterol/blood sugar^1^ |  |  |  |  | 30 | 27% |
| Learn blood pressure^1^ |  |  |  |  | 24 | 22% |
| Improve cholesterol | 39 | 39% | 32 | 32% | 10 | 9% |
| Achieve healthy weight | 12 | 12% | 19 | 19% | 12 | 11% |
| Move more | 22 | 22% | 18 | 18% | 27 | 25% |
| Improve blood sugar | 10 | 10% | 9 | 9% | 6 | 5% |
| Improve blood pressure | 10 | 10% | 8 | 8% | 9 | 8% |
| Stop smoking | 6 | 6% | 8 | 8% | 6 | 5% |
| Drink less alcohol | 6 | 6% | 6 | 6% | 5 | 5% |
| None | 20 | 20% | 22 | 22% | 28 | 25% |
| Actions taken | 60 | 62% | 56 | 57% | 51 | 46% |

F2F= Face-to-face health check; DHC = digital health check

^1^ These recommendations were only relevant to DHC where respondents did not always know their physical measurement data

**Table S7: Completion of physical measurement data in digital health check (DHC) : N = 1189 DHC completers**

|  | Known at DHC | | Updated | | Not known or updated | |
| --- | --- | --- | --- | --- | --- | --- |
|  | N | % | N | % | N | % |
| Height & weight | 1151 | 97% | 36 | 3% | 2 | <1% |
| Blood pressure | 274 | 23% | 30 | 3% | 884 | 74% |
| Cholesterol | 82 | 7% | 32 | 3% | 1075 | 90% |
| HbA1c | 50 | 4% | 29 | 2% | 1110 | 93% |

DHC = digital health check

**Table S8: Completion of physical measurements in digital health check among survey participants (results from six month survey)**

|  | Completed DHC (N=110) | | Physical measurements recommended  (N=50) |
| --- | --- | --- | --- |
|  | N | % | % |
| Home blood test | 12 | 11% | 20% |
| Pharmacy/leisure centre | 17 | 15% | 28% |
| Blood pressure kiosk | 3 | 3% | 5% |
| None | 78 | 71% | 47% |

DHC = digital health check

**Table S9: Service users identified as high risk by health check type**

|  | F2F | | | DHC | | |
| --- | --- | --- | --- | --- | --- | --- |
|  | Total | N | % | Total | N | % |
| **All** | | | | | | |
| High CVD risk^1^ | 292 | 42 | 14% | 1189 | 207 | 17% |
| High diabetes risk^2^ | 292 | 131 | 45% | 1189 | 355 | 30% |
| High CVD and high diabetes risk | 292 | 27 | 9% | 1189 | 99 | 8% |
| **Those with complete physical measurements only** | | | | | | |
| High CVD risk^1^ | 287 | 42 | 15% | 78 | 13 | 17% |
| High diabetes risk^2^ | 254 | 115 | 45% | 108 | 34 | 31% |
| High CVD and high diabetes risk | 249 | 24 | 10% | 50 | 5 | 10% |

F2F = Face-to-face health check; DHC = digital health check; CVD = cardiovascular disease

^1^ QRISK3 score >10

^2^ QDiabetes score > 5.6

**Table S10: Odd ratios of CVD and diabetes high risk for DHC versus F2F, adjusted for demographics**

|  |  | | | | | |
| --- | --- | --- | --- | --- | --- | --- |
|  | Moderate/High CVD risk | | | High Diabetes risk | | |
|  | n | OR | p-value | n | OR | p-value |
| Unadjusted | 1481 | 1.25 | 0.216 | 1481 | 0.52 | <0.005 |
| Adjusted^1^ | 1445 | 0.95 | 0.852 | 1445 | 0.45 | <0.005 |
| Adjusted^1^; physical measures provided | 361 | 0.68 | 0.477 | 357 | 0.99 | 0.982 |

^1^ Adjusted for age, sex, ethnicity, Index of Multiple Deprivation, smoking status and BMI category

**Table S11: Recommended GP follow-up and outcomes for Digital Health Check (DHC)**

|  |  | N | % |
| --- | --- | --- | --- |
| Recommended GP follow-up | |  |  |
|  | Non-urgent | 679 | 56% |
|  | Urgent | 14 | 1% |
| Chose GP follow-up as health priority | |  |  |
|  | % of all | 282 | 32% |
|  | % of those with non-urgent GP recommendation | 277 | 55% |
|  | % of those with urgent GP recommendation | 9 | 90% |
| Attended GP appointment^1^ | |  |  |
|  | % of all | 585 | 48% |
|  | % of those with non-urgent GP recommendation | 358 | 53% |
|  | % of those with urgent GP recommendation | 7 | 50% |

^1^ These include all GP appointments within the time frame, and are not necessarily linked to the health check.

**Table S12: GP Follow-ups by health check type allocated/completed**

|  | None  N=6913 | | F2F  N=305 | | DHC  N=1189 | | DHC->F2F  N=198 | |
| --- | --- | --- | --- | --- | --- | --- | --- | --- |
| **All** | | | | | | |  | |
| Appointments | 1427 | 21% | 264 | 87% | 560 | 47% | 177 | 89% |
| Medications | 1163 | 17% | 144 | 47% | 457 | 38% | 100 | 51% |
| Referrals | 561 | 8% | 131 | 43% | 236 | 20% | 79 | 40% |
| **Those at high risk of CVD^1^** | | | **N=42** | | **N=207** | |  | |
| Appointments |  |  | 36 | 86% | 101 | 49% |  |  |
| Medications |  |  | 27 | 64% | 101 | 49% |  |  |
| Referrals |  |  | 23 | 55% | 50 | 24% |  |  |
| **Those at high risk of diabetes^2^** | | | **N=131** | | **N=355** | |  | |
| Appointments |  |  | 118 | 90% | 195 | 55% |  |  |
| Medications |  |  | 67 | 51% | 169 | 48% |  |  |
| Referrals |  |  | 71 | 54% | 91 | 26% |  |  |

F2F = Face-to-face health check; DHC = digital health check

^1^ QRISK3 score >10

^2^ Qdiabetes score > 5.6

Note: These include all appointments, medications and referrals within the time frame, and are not necessarily as a result of the health check. Overall rates for non-health check completers are shown for comparison, but CVD/diabetes risk is not known for these users.

Too few DHC-> F2F users were identified as at high risk of CVD/diabetes risk to report.

**Table S13: F2F and DHC estimated cost data used in Table 2**

|  |  | | **Study** | | | | **Potential scale-up scenario** | | |
| --- | --- | --- | --- | --- | --- | --- | --- | --- | --- |
|  |  | | **N** | | **Total cost(£)** | **Unit cost (£)** | **N^1^** | **Total cost (£)** | **Unit cost (£)** |
| **F2F** |  | |  | |  |  |  |  |  |
| Uptake | | Identification^2^ | | 3,000 | 1,977 | 0.66 | 53,500 | 5,350 | 0.10 |
|  | | Invitation^3^ | | 3,000 | 6,833 | 2.28 | 53,500 | 71,690 | 1.34 |
| **DHC** | |  | |  |  |  |  |  |  |
| Uptake and assessment | | Identification^2^ | | 6,000 | 3,953 | 0.66 | 53,500 | 5,350 | 0.10 |
|  | | Invitation^3^ | | 5,705 | 11,141 | 1.95 | 50,870 | 68,165 | 1.34 |
|  | | Digital assessment^4^ | | 1,215 | 10,550 | 8.68 | 10,834 | 42,200 | 3.90 |
|  | | Result and data transfer^5^ | | 1,215 | 3,750 | 3.09 | 10,834 | 3,250 | 0.30 |
| Lifestyle support | | Digital lifestyle support^4^ | | 714 | 10,550 | 14.78 | 6,367 | 42,200 | 6.63 |
|  | | Clinical lifestyle support^6^ | | 714 | 2,250 | 3.15 | 6,367 | 1,910 | 0.30 |
| Physical measures recorded and updated | | Home finger prick tests^7^ | | 102 | 10,212 | 100.11 | 543 | 15,578 | 28.68 |
|  | | Leisure centres^8^ | | 20 | 729 | 36.45 | 178 | 3,670 | 20.58 |
|  | | Pharmacies^9^ | | n/a | 2,000 | n/a | 0 | 0 | 0.00 |
|  | | GP surgeries^9^ | | n/a | 3,250 | n/a | 0 | 0 | 0.00 |
|  | | Kiosks^10^ | | 357 | 1,489 | 4.17 | 3,183 | 13,274 | 4.17 |
|  | | physical measures updating^4^ | | 84 | 10,550 | 125.60 | 749 | 42,200 | 56.34 |

^1^ Scale-up: 107,000 individuals 40-74 years, targeting 53,500 individuals in each pathway over 4 years.

^2^ Study: local data allocated pro-rata between F2F and DHC users including assumed 20% (£1,500) of GP Federation costs. Scale-up: Identifying unit cost assumed £0.10 per individual;

^3^ Study: local data obtained from resource-used data. Scale-up: invitation cost £1.34 per individual from Office for Health Improvement & Disparities (2021).

^4^ Study: local cost data including hosting the service, ongoing support, configuration and testing, and bug fixes totalling £31,650 have been divided equally between digital assessment, digital lifestyle support and digital biometric data updating. Scale-up: assumed to be the same total cost per 12-month period for each of the four years.

^5^ Study: local data assumed that this was 50% of GP Federation cost. Scale-up: assumed unit cost of £0.30 per user.

^6^ Study: local data assumed that 30% of GP Federation cost. Scale-up: assumed unit cost of £0.30 per user.

^7^ Study: local data; 288 kits were sent with a unit cost of £28.08, 102 were returned, processed, and results sent with a unit cost of £20.83, and so the total cost per home finger prick test result obtained was £100.11. Scale-up: assumed a proportional activity based on Study data in the number of kits sent (1,534) and kits returned and processed (543) with a unit cost of £15 per kit processed.

^8^ Study: local data; 20 biometrics measurements conducted at £15 per check and £429 for contracts, setting, and training. Scale-up: assumed proportional activity based on Study data, cost per measurement £15 and £1,000 for contracts, setting, and training.

^9^ Study: local data; £2,000 for contracts, setting, and training at pharmacies. £3,250 for GP practice participation. Data on biometric measurements were not available. Scale-up: assumed no cost or activity in the absence of Study activity data.

^10^ Study: local data and assumed that 50% of the 714 individuals completed a biometric measurement in kiosks. Scale-up: assumed proportional activity based on Study data assumption, and associated unit cost.

**Table S14: Costs associated with identifying those at high-risk by pathway**

| **Pathway** | **High-risk service users**^1^ | | | **Pathway total cost (£)** | **Cost per high-risk individual (£)** |
| --- | --- | --- | --- | --- | --- |
| F2F | High-risk | 4% | 128 | 47,215 | 323 |
|  | Pseudo high-risk | 1% | 18 | 47,215 | 323 |
| DHC | High-risk | <1% | 23 | 124,237 | 5402 |
|  | Pseudo high-risk | 7% | 440 | 96,008 | 189 |

F2F = Face-to-face health check; DHC = digital health check

^1^ High-risk users are those identified from complete physical measurement data, and ‘pseudo-high-risk’ users are those identified where their data was incomplete, and population-based estimates of physical measures were used in calculating QRISK3 and QDiabetes scores

Denominator equals the number of users allocated in each pilot pathway (F2F N = 3000; DHC N = 6000)
