# Supplementary material for: A service evaluation of the uptake and effectiveness of a digital delivery of the NHS health check service
Source: BMJ Open. 2024 Nov 9;14(11):e091417. doi: 10.1136/bmjopen-2024-091417 (PMC11552007; doi:10.1136/bmjopen-2024-091417)
Supplement: online supplemental file 2 [file bmjopen-14-11-s002.pdf]

NHS Health Check User Survey

Patient experience of the Southwark NHS Health Check service

Thank you for your interest in this project. The purpose of this survey is to help us understand what you think about the NHS Health Checks. You have been asked to take part in this survey because you were invited to complete an NHS Health Check [F2F ONLY] /Digital Health Check [DHC ONLY] within the last 6 months via SMS or letter - it does not matter whether or not you chose to complete a Health Check, we would still like to hear from you in this survey.

This survey should take **no more than 10 minutes** to complete.

At the end of the survey, you will be asked if you would like to be entered into a prize draw to win one of ten **£50 shopping vouchers**. If so, please provide your name and contact details.

Southwark Council is working with a team of researchers at the University of Bristol, who will use data collected in this survey to help improve the Health Check and other services.

How will we use information about you?

We will need to use information from you for research projects.

This information will include your name and contact details. People will use this information to do the research or to check your records to make sure that the research is being done properly.

People who do not need to know who you are will not be able to see your name or contact details. Your data will have a code number instead.

We will keep all information about you safe and secure.

Once we have finished the study, we will keep some of the data so we can check the results. We will write our reports in a way that no-one can work out that you took part in the study.

What are your choices about how your information is used?

- You can stop being part of the study at any time, without giving a reason, but we will keep information about you that we already have.
- We need to manage your records in specific ways for the research to be reliable. This means that we won't be able to let you see or change the data we hold about you.

Where can you find out more about how your information is used?

You can find out more about how we use your information

- at www.hra.nhs.uk/information-about-patients/
- by sending an email to data-protection@bristol.ac.uk

By completing and submitting this survey, you are consenting to us using your responses to understand more about the Health Check service and how it could be improved. For more

information on how your data will be managed and used, please see our Privacy Statement <link to pdf privacy statement>.

Please click ‘next’ to continue.

Your Health Check invitation

1. After receiving your Health Check invitation, did you complete a Health Check?
   a. Yes, I had a Health Check at my GP practice
   b. [DHC ONLY] Yes, I completed a digital Health Check (using the website)
   c. No, I did not have a Health Check

The Health Check at a GP practice

2. Please tell us why you decided to have a Health Check at your GP practice.

   Select as many as apply

   - [ALLOCATED TO F2F ONLY]I was interested to know more about my health
   - I had some concerns/questions about my health that I wanted to discuss with a health professional
   - I needed to visit the GP practice for another reason (e.g., pick up a prescription), so had my Health Check at the same time
   - [ALLOCTED TO DHC ONLY] I dislike or find it difficult doing things online
   - My GP practice is friendly and I enjoy seeing people there
   - My GP practice is easy for me to get to
   - Other *[if selected: “Please state”]*

3. Did you have any problems in completing your Health Check (e.g., difficulties getting an appointment at your GP practice)?
   a. Yes
   b. No
   [If yes] Please tell us what problems you had.

4. Based on the results of your Health Check, were you advised to do any of the following: *[Select as many as apply]*

   - Move more

   [if selected, ask: “Were you provided with any information or referred to services that would help you do this?”]
     - Yes
     - No

   [if selected, ask: “Since your Health Check, have you taken any action to move more?”]
     - Yes
       [if selected, ask: “What action did you take?”]
       Select as many as apply
       - Walking/running/cycling more to get to places (e.g., to work)
       - Joined a gym/go to a gym more often

        - Joined an exercise class or group
        - Started doing/doing more exercise at home (e.g., online workouts)
        - Looked for information on moving more (e.g., online)
        - Other *[if selected: "Please state"]*
    - No
- Stop smoking
  [if selected, ask: "Were you provided with any information or referred to services that would help you do this?"]
    - Yes
    - No
      [if selected, ask: "Since your Health Check, have you taken any action to stop smoking?"]
    - Yes
      [if selected, ask : "What action did you take?"]
        - *Select as many as apply*
        - Made an appointment to speak to a **stop smoking** advisor
        - Attended an appointment with a **stop smoking** advisor
        - Stopped smoking
        - Started cutting down the number of cigarettes you smoke
        - Started using nicotine gum/patches
        - Switched to using e-cigarettes
        - Looked for information on stopping smoking (e.g., online)
        - Other *[if selected: "Please state"]*
    - No
- Drink less alcohol
  [If selected, ask: "Were you provided with any information or referred to services that would help you do this?"]
    - Yes
    - No
  [if selected, ask: "Since your Health Check, have you taken any action to drink less alcohol?"]
    - Yes
        - *[if selected, ask : "What action did you take?"]*
        - *Select as many as apply*
        - Made an appointment to speak to your GP about your alcohol intake
        - Attended an appointment with your GP about your alcohol intake
        - Started cutting down the amount of alcohol you drink
        - Joined a support group for people with alcohol dependency
        - Looked for information on cutting down on alcohol (e.g., online)
        - Other *[if selected: "Please state"]*
    - No
- Pursue a healthy weight

[*if selected, ask: "Were you provided with any information or referred to services that would help you do this?"]*
    - Yes
    - No

[if selected, ask: "Since your Health Check, have you taken any action to pursue a healthy weight?"]

- Yes

  [if selected, ask: "What action did you take?"]

  Select as many as apply
    - Made an appointment to speak to your GP about your weight
    - Attended an appointment with your GP about your weight
    - Attended a weight management programme (e.g., Weight Watchers)
    - Looked for information on weight (e.g., online)
    - Made changes to your diet
    - Started moving/exercising more
    - Other *[if selected: "Please state"]*
- No

  [if selected, ask: "Since your Health Check, have you lost any weight?"]
- Yes

  [if selected: "Please tell us how much weight you have lost"]
- No

- Improve your blood pressure

  [*if selected, ask: "Were you provided with any information or referred to services that would help you do this?"]*
    - Yes
    - No

  [if selected, ask: "Since your Health Check, have you taken any action to improve your blood pressure?"]
    - Yes

      [if selected, ask: "What action did you take?"]

      Select as many as apply
        - Made an appointment to speak to your GP about your blood pressure
        - Attended an appointment with your GP about your blood pressure
        - Taking prescribed medication (e.g., ramipril, losartan, amlodipine)
        - Looked for information on managing your blood pressure (e.g., online)
        - Made changes to your diet
        - Started moving/exercising more
        - Other *[if selected, ask : "Please state"]*
    - No
- Improve your cholesterol levels

  [*if selected, ask: "Were you provided with any information or referred to services that would help you do this?"]*
    - Yes
    - No

  [if selected, ask: "Since your Health Check, have you taken any action to improve your cholesterol levels?"]
    - Yes

      [if selected, ask: "What action did you take?"]

      Select as many as apply
        - Made an appointment to speak to your GP about your cholesterol levels
        - Attended an appointment with your GP about your cholesterol levels
        - Taking prescribed medication (e.g., statins)
        - Looked for information on managing cholesterol levels (e.g., online)

        - Made changes to your diet
        - Started moving/exercising more
        - Other *[if selected, ask : "Please state"]*
    - No
- Improve your blood sugar level

  [*if selected, ask: "Were you provided with any information or referred to services that would help you do this?"]*
    - Yes
    - No

      [if selected, ask: "Since your Health Check, have you taken any action to improve your blood sugar levels?"]
    - Yes

      [if selected, ask: "What action did you take?"]

      Select as many as apply
        - Made an appointment to speak to your GP about your blood sugar levels
        - Attended an appointment with your GP about your blood sugar levels
        - Taking prescribed medication (e.g., metformin)
        - Looked for information on managing blood sugar levels (e.g., online)
        - Made changes to your diet
        - Started moving/exercising more
        - Other *[if selected, ask : "Please state"]*
    - No
- Other

  [if selected, ask: "What were you advised to do?"]

  [if selected, ask: "Since your Health Check, have you taken any action towards this?"]
    - Yes

      [if selected, ask : "What action did you take?"]
    - No
- I was not advised to do any of these.

5. If, as a result of your Health Check, you received any information or were referred to services to help you improve your health, how satisfied were you with this information/these services?

- Not satisfied - most of the services and information did not meet my expectations.
- Partly satisfied - some of the services and information met my expectations.
- Satisfied - most of the services and information met my expectations.
- Very satisfied – the services and information exceeded my expectations.

  [If selecting any of these 4 options, then ask: "Please tell us what was satisfactory/dissatisfactory?"]

  [If selecting any of these 4 options, then ask: "Is there any more information or support that you would have liked to receive?"]
- I did not receive any information or referrals.

  [if selected, ask: "Is there any information or support that you would have liked to receive?"]

6. People are encouraged to have a NHS Health Check every 5 years.
    a. A face to face NHS Health Check involves going to your GP clinic for a 25 minute session where you answer questions about your health and lifestyle (exercise,

alcohol consumption) and have some measurements taken (e.g. blood pressure, blood sugar). You get results and recommendations for either treatment or changes you can make to your lifestyle to improve your health.

b. In the future there may be an option to complete a NHS Health Check online – a 'Digital Health Check' - this would involve answering the same questions as the face to face health check via a website/app, with the option of doing a blood test with a kit sent to your home or visiting a pharmacy/leisure centre for your measurements. You will also get results and recommendations for either treatment or changes you can make to your lifestyle to improve your health.

7. For your next Health Check, which of the following do you think you will choose?
    a. A Health Check at my GP practice
    b. A digital Health Check (using the website)
    c. I do not think I will have a Health Check

    [For all options, ask: "Please explain your choice."]

8. "On a scale of 0-10, how likely is it that you would recommend the NHS Health Check service at a GP practice to your friends or family?"
    0-10, 0 Not likely, 10 is extremely likely

The digital Health Check

[DHC COMPLETED]

2. Please tell us why you decided to complete a digital Health Check.
   Select as many as apply
   - I could complete it a time that suited me/It was easier to fit in around work and other commitments
   - I find it uncomfortable talking about my health with health professionals
   - It is hard to get an appointment at my GP practice
   - I did not realise that I could have a Health Check at my GP practice
   - Other *[if selected: "Please state"]*

3. Did you have any problems in completing your digital Health Check (e.g., issues with the website)?
   - Yes
     [If yes] Please tell us what problems you had.
   - No

4. Based on your results in the digital Health Check, were you recommended to do either of the following?
   - complete a home blood test (to measure your cholesterol and blood sugar levels)
     - Yes
       [if selected, ask: "And did you do a home blood test?"]
       - Yes
       - No
         [if selected: "What stopped you or made you decide not to do a home blood test?"]
         Select all that apply
         - I ordered the test but forgot to complete it
         - I did not think I needed a blood test
         - I do not like blood
         - I do not know how to complete a blood test
         - Other *[if selected: "Please state"]*
     - No

   - visit a pharmacy or leisure centre to have your height, weight and blood pressure measured
     - Yes
       [if selected, ask: "And did you get measured at a pharmacy or leisure centre?"]
       - Yes
       - No
         [if selected: "What stopped you or made you decide not to get measured?"]
         Select all that apply
         - I booked an appointment but forgot/was unable to go
         - It is hard for me to get to a pharmacy/leisure centre
         - I did not think I needed to be measured

        - Other
        - *[if selected: "Please state"]*
    - No

5. Based on the results of your Health Check, were you advised to do any of the following: *[Select as many as apply]*

- Move more
[if selected, ask: "Were you provided with any information or referred to services that would help you do this?"]
    - Yes
    - No
[if selected, ask: "Since your Health Check, have you taken any action to move more?"]
    - Yes
      [if selected, ask: "What action did you take?"]
      Select as many as apply
        - Walking/running/cycling more to get to places (e.g., to work)
        - Joined a gym/go to a gym more often
        - Joined an exercise class or group
        - Started doing/doing more exercise at home (e.g., online workouts)
        - Looked for information on moving more (e.g., online)
        - Other *[if selected: "Please state"]*
    - No
- Stop smoking
  [if selected, ask: "Were you provided with any information or referred to services that would help you do this?"]
    - Yes
    - No
      [if selected, ask: "Since your Health Check, have you taken any action to stop smoking?"]
    - Yes
      [if selected, ask : "What action did you take?"]
        - *Select as many as apply*
        - Made an appointment to speak to a **stop smoking** advisor
        - Attended an appointment with a **stop smoking** advisor
        - Stopped smoking
        - Started cutting down the number of cigarettes you smoke
        - Started using nicotine gum/patches
        - Switched to using e-cigarettes
        - Looked for information on stopping smoking (e.g., online)
        - Other *[if selected: "Please state"]*
    - No
- Drink less alcohol
  [If selected, ask: "Were you provided with any information or referred to services that would help you do this?"]
    - Yes

- No

[if selected, ask: "Since your Health Check, have you taken any action to drink less alcohol?"]

- Yes
    - *[if selected, ask : "What action did you take?"]*
    - *Select as many as apply*
    - Made an appointment to speak to your GP about your alcohol intake
    - Attended an appointment with your GP about your alcohol intake
    - Started cutting down the amount of alcohol you drink
    - Joined a support group for people with alcohol dependency
    - Looked for information on cutting down on alcohol (e.g., online)
    - Other *[if selected: "Please state"]*
- No

- Improve your mental wellbeing

  [*if selected, ask: "Were you provided with any information or referred to services that would help you do this?"]*
    - Yes
    - No

    [if selected, ask: "Since your Health Check, have you taken any action to improve your mental wellbeing?"]

    - Yes

      [if selected, ask: "What action did you take?"]
      Select as many as apply
        - Made an appointment to speak to your GP about your mental health
        - Attended an appointment with your GP about your mental health
        - Attended talking therapy (e.g., psychotherapy, counselling)
        - Phoned the NHS mental health line or other telephone support service
        - Looked for information on mental health (e.g., online)
        - Other *[if selected: "Please state"]*
    - No
- Pursue a healthy weight

[*if selected, ask: "Were you provided with any information or referred to services that would help you do this?"]*

- Yes
- No

[if selected, ask: "Since your Health Check, have you taken any action to pursue a healthy weight?"]

- Yes

  [if selected, ask: "What action did you take?"]
  Select as many as apply
    - Made an appointment to speak to your GP about your weight
    - Attended an appointment with your GP about your weight
    - Attended a weight management programme (e.g., Weight Watchers)
    - Looked for information on weight (e.g., online)
    - Made changes to your diet
    - Started moving/exercising more
    - Other *[if selected: "Please state"]*

        - No
          [if selected, ask: "Since your Health Check, have you lost any weight?"]
            - Yes
              [if selected: "Please tell us how much weight you have lost"]
            - No

- Improve your blood pressure
  [*if selected, ask: "Were you provided with any information or referred to services that would help you do this?"]*
    - Yes
    - No

  [if selected, ask: "Since your Health Check, have you taken any action to improve your blood pressure?"]
    - Yes
      [if selected, ask: "What action did you take?"]
      Select as many as apply
        - Made an appointment to speak to your GP about your blood pressure
        - Attended an appointment with your GP about your blood pressure
        - Taking prescribed medication (e.g., ramipril, losartan, amlodipine)
        - Looked for information on managing your blood pressure (e.g., online)
        - Made changes to your diet
        - Started moving/exercising more
        - Other *[if selected, ask : "Please state"]*
    - No
- Improve your cholesterol levels
  [*if selected, ask: "Were you provided with any information or referred to services that would help you do this?"]*
    - Yes
    - No

  [if selected, ask: "Since your Health Check, have you taken any action to improve your cholesterol levels?"]
    - Yes
      [if selected, ask: "What action did you take?"]
      Select as many as apply
        - Made an appointment to speak to your GP about your cholesterol levels
        - Attended an appointment with your GP about your cholesterol levels
        - Taking prescribed medication (e.g., statins)
        - Looked for information on managing cholesterol levels (e.g., online)
        - Made changes to your diet
        - Started moving/exercising more
        - Other *[if selected, ask : "Please state"]*
    - No
- Improve your blood sugar level
  [*if selected, ask: "Were you provided with any information or referred to services that would help you do this?"]*
    - Yes
    - No

[if selected, ask: "Since your Health Check, have you taken any action to improve your blood sugar levels?"]

- Yes

  [if selected, ask: "What action did you take?"]
  Select as many as apply

  - Made an appointment to speak to your GP about your blood sugar levels
  - Attended an appointment with your GP about your blood sugar levels
  - Taking prescribed medication (e.g., metformin)
  - Looked for information on managing blood sugar levels (e.g., online)
  - Made changes to your diet
  - Started moving/exercising more
  - Other *[if selected, ask : "Please state"]*
- No

- Other

  [if selected, ask: "What were you advised to do?"]
  [if selected, ask: "Since your Health Check, have you taken any action towards this?"]

  - Yes

    [if selected, ask : "What action did you take?"]
  - No
- I was not advised to do any of these.

6. If, as a result of your Health Check, you received any information or were referred to services to help you improve your health, how satisfied were you with this information/these services?
   - Not satisfied - most of the services and information did not meet my expectations.
   - Partly satisfied - some of the services and information met my expectations.
   - Satisfied - most of the services and information met my expectations.
   - Very satisfied – the services and information exceeded my expectations.

     [If selecting any of these 4 options, then ask: "Please tell us what was satisfactory/dissatisfactory?"]
     [If selecting any of these 4 options, then ask: "Is there any more information or support that you would have liked to receive?"]
   - I did not receive any information or referrals.

     [if selected, ask: "Is there any information or support that you would have liked to receive?"]

7. People are encouraged to have a NHS Health Check every 5 years.
   a. A face to face NHS Health Check involves going to your GP clinic for a 25 minute session where you answer questions about your health and lifestyle (exercise, alcohol consumption) and have some measurements taken (e.g. blood pressure, blood sugar). You get results and recommendations for either treatment or changes you can make to your lifestyle to improve your health.
8. In the future the '**Digital Health Check**' - like the one you completed - may be an option. This would involve answering the same questions as the face to face health check via a website/app, with the option of doing a blood test with a kit sent to your home or visiting a pharmacy/leisure centre for your measurements. You will also get results and

recommendations for treatment or changes you can make to your lifestyle to improve your health. For your next Health Check, which of the following do you think you will choose?

a. A Health Check at my GP practice
b. A digital Health Check (using the website)
c. I do not think I will have a Health Check

[For all options, ask: "Please explain your choice."]

9. "On a scale of 0-10, how likely is it that you would recommend the Digital NHS Health Check service to your friends or family?"
0-10, 0 Not likely, 10 is extremely likely

[NO HEALTH CHECK COMPLETED]

2. Please tell us what stopped you or made you decide not to have a Health Check.
   Select all that apply
   - I booked a Health Check at my GP practice but was unable to attend
   - I tried to book a Health Check at my GP practice but could not get an appointment
   - I started completing the digital Health Check but ran out of time/was unable to finish
   - I dislike or find it difficult doing things online
   - I did not think I needed a Health Check
   - Other *[if selected: "Please state"]*

3. Please tell us if there is anything that would encourage or make it easier for you to have a Health Check.
   Select all that apply
   - More information on what is involved in a Health Check and why I should have one
   - Help with child care/travel to attend a Health Check at my GP practice
   - Health Check appointments at my GP practice available in early mornings/late evenings
   - Help completing a digital Health Check
   - An incentive (e.g., shopping vouchers for completing a Health Check)
   - Other *[if selected: "Please state"]*

4. People are encouraged to have a NHS Health Check every 5 years.
   a. A face to face NHS Health Check involves going to your GP clinic for a 25 minute session where you answer questions about your health and lifestyle (exercise, alcohol consumption) and have some measurements taken (e.g. blood pressure, blood sugar). You get results and recommendations for either treatment or changes you can make to your lifestyle to improve your health.
   b. In the future there may be an option to complete a NHS Health Check online – a 'Digital Health Check' - this would involve answering the same questions as the face to face health check via a website/app, with the option of doing a blood test with a kit sent to your home or visiting a pharmacy/leisure centre for your measurements. You will also get results and recommendations for either treatment or changes you can make to your lifestyle to improve your health.

5. When you are next invited for a Health Check, which of the following do you think you will choose?
   a. A Health Check at my GP practice
   b. A digital Health Check (using the website)
   c. I do not think I will have a Health Check

   [For all options, ask: "Please explain your choice."]

6. "On a scale of 0-10, how likely is it that you would recommend your friends or family to have a Health Check?"
   0-10, 0 Not likely, 10 is extremely likely

Final page
[ALL PARTICIPANTS]

Thank you for completing this survey – your responses are really important and will help us to improve health services.

If you would like to be entered in the prize draw to win a £50 shopping voucher, please provide your name and email address, so that we can contact you if you win.

Name:

Email:
