# Supplementary material for: A service evaluation of the uptake and effectiveness of a digital delivery of the NHS health check service
Source: BMJ Open. 2024 Nov 9;14(11):e091417. doi: 10.1136/bmjopen-2024-091417 (PMC11552007; doi:10.1136/bmjopen-2024-091417)
Supplement: Supplementary file 4 [file bmjopen-2024-091417.reviewer_comments.pdf]

PEER REVIEW HISTORY

BMJ Open publishes all reviews undertaken for accepted manuscripts. Reviewers are asked to complete a checklist review form and are provided with free text boxes to elaborate on their assessment. These free text comments are reproduced below.

ARTICLE DETAILS

Title (Provisional)

A service evaluation of the uptake and effectiveness of a digital delivery of the NHS Health Check service

Authors

Salway, Ruth E; Sillero-Rejon, Carlos; Forte, Chloe; Grey, Lis; Jessiman, Tricia; McLeod, Hugh; Harkes, Rebecca; Stokes, Paul; De Vocht, Frank; Campbell, Rona; Jago, Russ

---

VERSION 1 - REVIEW

**Reviewer**	**1**
**Name**	**Al-Durra, Mustafa**
**Affiliation**	**University of Toronto, Centre for Global eHealth Innovation, Techna Institute, University Health Network**
**Date**	**26-Aug-2024**
**COI**	**n/a**

This paper examined the uptake, effectiveness and costs of a digital version of the NHS Health Check compared to the standard face-to-face NHS Health Check for a sample of 9000 patients, aged 40-74, in Southwark, England between January and April 2023. The digital version of the NHS Health Check, and the focus of this study, is to identify individuals with increased risk of cardiovascular disease and/or diabetes.

Given the burden, and prevalence, of these two conditions in the UK population and globally, this study provides quantitative analysis on how digital health can contribute to improving preventative health, and promoting behavior change for a large cohort of patients.

The digital health industry has witnessed many advancements in the recent years, from remote patients monitoring with wearable devices, to AI-powered virtual health assistant, to multi-modal biomedical AI that could help with identifying individuals with increased risk of cardiovascular disease and/or diabetes in more effective and innovative ways. This is evidenced by a recent scoping review (Singh et al. 2024, https://www.thelancet.com/journals/eclinm/article/PIIS2589-5370(24)00239-6/fulltext),

that concluded “Intricate pattern extraction from large, complicated genetic datasets is a skill that AI algorithms excel at, allowing for accurate disease diagnosis and CVD risk prediction”.

In 2016, the World Health Organization published a maturity matrix for evaluating digital health interventions (Table 1.1 in https://www.who.int/publications/i/item/9789241511766). The DHC arm of this study can be categorized as ‘Early’ in the stage of maturity, for a technology that is no longer state-of-the-art. The readers may be underwhelmed by the lack of innovation in the digital health component in this study, namely being limited to a website with self-assessment forms. Furthermore, this research intervention needs to be evaluated in non-research (uncontrolled) setting first, i.e. ‘Mild’ stage of maturity, before advancing to scale-up to half of the Southwark population.

Reviewer Comments:

Registration

- Risk of registration Bias. What is the trial registration number? When was the trial registered, and when was the first patient enrolled? Where can I find the trial registration protocol to measure reporting and registration bias? Please follow the ICMJE and WHO guidelines for prospective trial registration (Al-Durra et al. BMJ 2020: https://www.bmj.com/content/369/bmj.m982.long).

Abstract section\Intervention & Design:

- “Those invited to DHC were able to choose F2F if they preferred”. Why did the study only offer DHC patients to switch to F2F, and not also offer F2F patients to switch to DHC? Would this limitation have introduced a sampling bias in the DHC cohort, being that those remaining DHC patients are more intentional and perhaps more committed/interested in the DHC, compared to those how had no choice to leave F2F? The authors need to explain why they continue to consider this trial design as ‘randomized’ vs ‘quasi-randomized’?

Abstract section\Outcomes:

- Given that the study had access to GP records of 6000 patients, why did the design only include appointment, medication and referral data? In other words, why were conditions, lab results, and procedures not included in this analysis?

Abstract\Results:

- Because DHC is a digital intervention, why did the study excluded patients who had moved away?

- Risk of reporting bias: The results section starts with reporting on the DHC “uptake”, whereas the outcomes section never mentioned the word “uptake”? To control the risk of reporting bias, the authors need to be explicit in defining/detailing the primary (and secondary if any) outcome measures, and report accordingly in the result section.

- Risk of reporting bias: In the outcome section, the cost of development was included in the primary outcome, but the result section did not report on the development cost?

- Risk of cofounding factors: Because DHC patients had the opportunity to switch to F2F if they wanted to, it's likely that those patients who chose to stay in DHC would favor remote interactions. Could those preferences have attributed to lower on appointments and referrals? Authors should also indicate in the abstract if it was significantly, or not significantly, lower.

- Because the study has access to the full GP records, should the comparison consider the evaluation of appointments, medication and referral, before and after the DHC intervention.

- "Compared to DHC costs of £68.48 for health assessment only, £134.46 including the support section, and £1479.01 per user with completed physical measures". Please, refine/simplify this sentence. It is clear how much is the estimated total measurement and operation costs per user for DHC?

- Qualitative measure – it is important to learn more about the patient sentiment, in particular for those who chose to opt for F2F, and leave DHC? It will be insightful to learn if the reported lower appointments and referrals in the DHC cohort was, in part, because those DHC patients generally disengaged with in-person appointment/visits to the GP, and hence did not opt to go F2F and stay in DHC. The NIHR 2019 report (Informing NHS policy in 'digital-first primary care': a rapid evidence synthesis (nih.gov) addresses some of those concerns: "Although uptake of alternative consultation technologies in primary care was found to be low, it was unclear if this was due to GPs applying the use of alternatives selectively, patients not being aware of their availability or not wishing to use them, or problems with implementation. The authors also noted a lack of evidence relating to the roles of team members, such as reception staff, in delivering alternatives to the face-to-face consultation"

Introduction

- Risk of selective reporting bias: The authors draw a reference on how "Both CVD and type 2 diabetes are strongly associated with health inequalities" – is this topic relevant/addressed by this study? Is so, (1) please indicated in the study objectives?

- "…assess the extent to which the DHC is effective at engaging those groups that are under-represented in F2F". The design methods and results do not conform with this statement. Please clarify if the objective of the DHC design was based on the under-represented patient groups in F2F? If so, the inclusion criteria need to reflect that please.

- With respect to the 2018 rapid evidence synthesis study (Rodgers, M., et al. 2019), the findings indicated that users of digital health interventions are likely to be younger, female and have higher income and education levels. In this case, the authors should explain how they envisioned a digital health intervention, contrary to the findings of that 2018 study, will in fact benefit under-represented patients and address healthcare inequalities.

Methods\Study design

- It will be important to declare why 9000 patients were selected in this sample. Was it a form of convenience sampling?

- What was the rationale for the uneven split between the DHC and F2F cohorts, being 2:1 (6000:3000)? If it was bound to recruitment feasibility for DHC vs F2F, why did the split not remain even 3000 each?

- What is IRAS?

- “followed by an SMS reminder 2 days later (DHC) or one week later (F2F)”. It seems F2F is an existing program that makes it hard to change and align with this study design. Could the authors explain why the rational behind not sending DHC reminder after one week, instead of 2 days, to align with F2F?

Methods\Health check details

- What is “GP surgery”? And what’s the difference between ‘GP Surgery’ and ‘GP’?

- Figure1 “Overview of Face-to-Face (F2F) and Digital Health Check (DHC) pathways” is not readable and distorted when zoomed in. I therefore was not able to review it.

- “Those service users whose results”. What is the service the authors are referring to? Did the authors mean to say “Those users whose results…” instead?

- “Those users at very high risk were advised to contact their GP clinic urgently: those

who were very underweight”. Perhaps, the authors could rephrase for more clarity and simplicity.

Methods\Data

- “Those from the most deprived areas were defined as residing in areas form the lowest deprivation quintile”. Please, indicate and cite the SDOH scale that was used to identify the “most deprived” and “lowest deprivation quintile”. In table 1, there is a reference to IMD. What is IMD?. In table S1, there is a reference to index of multiple deprivation, please provide a citation and clear reference to the year and country in scope for that index.

- Please rephrase and elaborate on the limitation here, or perhaps add to the limitation section. “Note that due to limitations of the data, these relate to any appointments within the time frame and may not link directly to a health check”. What is the limitation? One which data, which group, and what is the magnitude and impact of this limitation to the reported findings of this study?

- “The user survey contained questions on respondent opinions of the health checks, motivation to attend health checks, satisfaction with services offered post health check, and any actions taken following health check completion”. It seems we had a missed opportunity to understand why 198 patients opted for F2F instead of DHC? It will be great to learn why those patients opted out for F2F?

Results\Table 2

- The development cost was estimated at £261,000. This is not a static cost. There will be an ongoing operational cost for hosting and technical support and maintenance. While this cost was accounted for within the context of this study, it will important to factor in the estimated cost to sustain, scale up the website for the larger number of users on a five-years basis – given that, as indicated by the authors, local Authorities are required to offer all eligible individuals an NHS Health Check every five years.

**Reviewer**	**2**
**Name**	**Kilańska, Dorota**
**Affiliation**	**Medical University of Lodz, Coordinated Care Department**
**Date**	**29-Aug-2024**
**COI**	**N/A**

Line 31-31 quotes are with or without spaces, it would be good to standardize, although it is only an editing error.

**Reviewer**	**3**
**Name**	**Lin, Simon**
**Affiliation**	**Symptoma GmbH, Science Department**
**Date**	**01-Sep-2024**
**COI**	**no competing interests**

The manuscript titled "An Evaluation of the Uptake and Effectiveness of a Digital Delivery of the NHS Health Check Service: a randomised controlled study" presents an investigation into the adoption and impact of a digital approach to a well-established public health intervention. With the growing emphasis on and urgent need for the digital delivery of health services, this study is a welcome contribution to the current literature by exploring how digital delivery methods might enhance or hinder the NHS Health Check program's reach and effectiveness. The study's relevance is underscored by its potential implications for public health policy contributing to the bedrock of evidence advocating for the swift uptake of more digital solutions.

In reviewing this manuscript, some aspects require further refinement to optimally render its outcomes' significance precisely:

1. In the methods section under the subsection "Data" you mention that you "...estimated the costs using resource and cost data supplied by Southwark staff." - Regarding this more

information would be needed to understand the underlying data. Who is the mentioned Southwark staff precisely, are they employees from a specific agency in Southwark? Where is the data from? What kind of data has been provided?

2. Further explanations or references regarding the "health priorities" would help better understand the potential added value to the individuals' health. What do those engulf exactly? How are they delivered concretely (text, videos, apps, gamification, etc.)?

3. One of the main limitations highlighted in the study has been the different recruitment processes for the F2F and DHC pathways. As such it would be important to characterize those further. Is there an estimation of the successfully delivered SMS messages as mobile phone numbers can be outdated in the EMIS or whatever registry extracted from? The same applies to the addresses the additional letters were sent to regarding the DHC.

Further were there any differences in the SMS reminders between the F2F and DHC pathways? Providing the message text as part of the supplemental material could be helpful regarding reproducibility and future improvements.

4. One of the main findings was the immature process of obtaining physical measures when missing for the DHC pathway. You describe that " Those users who did not provide physical measures were offered the option of finding out as one of their health priorities, and given choices and directions to obtain measures via pharmacies, leisure centres, kiosks, GP surgeries or a home finger-prick blood test kit". It will be critical to characterize the current processes better to understand the existing potential barriers for users to obtain such measures individually. Which steps have to be taken by users for each of those options (pharmacies, leisure centres, kiosks, GP surgeries, home finger-prick blood test kit) to receive their physical measurements?

Overall, the manuscript provides valuable insights into the potential benefits and challenges associated with introducing digital public health services. The study's findings are particularly pertinent for policymakers, healthcare providers, and researchers interested in digital health innovation and its role in preventive care. However, to strengthen the manuscript further, the aforementioned concerns should be addressed. Addressing these issues will enhance the manuscript's clarity, rigor, and overall contribution to the field. With these revisions, this study has the potential to make a meaningful contribution to accelerate the digital health strategies within the NHS and beyond.

VERSION 1 - AUTHOR RESPONSE

Reviewer: 1

This paper examined the uptake, effectiveness and costs of a digital version of the NHS Health Check compared to the standard face-to-face NHS Health Check for a sample of 9000 patients, aged 40-74, in Southwark, England between January and April 2023. The

digital version of the NHS Health Check, and the focus of this study, is to identify individuals with increased risk of cardiovascular disease and/or diabetes.
Given the burden, and prevalence, of these two conditions in the UK population and globally, this study provides quantitative analysis on how digital health can contribute to improving preventative health, and promoting behavior change for a large cohort of patients.

The digital health industry has witnessed many advancements in the recent years, from remote patients monitoring with wearable devices, to AI-powered virtual health assistant, to multi-modal biomedical AI that could help with identifying individuals with increased risk of cardiovascular disease and/or diabetes in more effective and innovative ways. This is evidenced by a recent scoping review (Singh et al. 2024, https://www.thelancet.com/journals/eclinm/article/PIIS2589-5370(24)00239-6/fulltext), that concluded "Intricate pattern extraction from large, complicated genetic datasets is a skill that AI algorithms excel at, allowing for accurate disease diagnosis and CVD risk prediction".

In 2016, the World Health Organization published a maturity matrix for evaluating digital health interventions (Table 1.1 in https://www.who.int/publications/i/item/9789241511766). The DHC arm of this study can be categorized as 'Early' in the stage of maturity, for a technology that is no longer state-of-the-art. The readers may be underwhelmed by the lack of innovation in the digital health component in this study, namely being limited to a website with self-assessment forms. Furthermore, this research intervention needs to be evaluated in non-research (uncontrolled) setting first, i.e. 'Mild' stage of maturity, before advancing to scale-up to half of the Southwark population.

We recognise that there has been lots of innovation in this space and that there are lots of new AI level type interventions that are bring developed. However, this was a pragmatic evaluation of a service that was provided by the National Health Service (NHS) in one London Borough. This is important as UK Health Policy is intended to roll-out this type of service. We have added a few sentences to the limitations section (lines 583-86) that recognises that there are newer / more advanced digital health interventions being developed and these may have further benefits but will need to be evaluated.

Reviewer Comments:

Registration
- Risk of registration Bias. What is the trial registration number? When was the trial registered, and when was the first patient enrolled? Where can I find the trial registration protocol to measure reporting and registration bias? Please follow the ICMJE and WHO guidelines for prospective trial registration (Al-Durra et al. BMJ 2020: https://www.bmj.com/content/369/bmj.m982.long).

As noted in our response to the editor, this was a service evaluation and not a clinical trial; we have changed the language throughout to make this clearer.

The study and protocol were pre-registered on the Open Science Framework (see line 46). We realise that it was not clear in the original manuscript that the reference in the methods was to the registration so we have updated the text at line 127-28:

> The study **protocol was pre-registered on the Open Science Framework [20], and this study** forms part of the...

Abstract section\Intervention & Design:
- "Those invited to DHC were able to choose F2F if they preferred". Why did the study only offer DHC patients to switch to F2F, and not also offer F2F patients to switch to DHC? Would this limitation have introduced a sampling bias in the DHC cohort, being that those remaining DHC patients are more intentional and perhaps more committed/interested in the DHC, compared to those how had no choice to leave F2F? The authors need to explain why they continue to consider this trial design as 'randomized' vs 'quasi-randomized'?

As this was a service evaluation, the primary comparison was between usual care (which is F2F with no digital option) versus the digital version in the format that would be rolled out if successful (DHC with the option to choose F2F if preferred). Please see our response to the editor's feedback above for further details, plus we have added the following clarifications before the objectives (line 112-15):

> The evaluation compares usual NHS Health Check procedure, a face-to-face Health Check (F2F), with the DHC, offered with a choice of F2F for those who prefer, in the form that would be implemented more widely if successful, that is as a complementary service to the existing programme.

The reviewer is correct that there may be differences in who chooses DHC. We have now clarified that our primary outcome was 'completion of any type of health check' and not 'completion of DHC/F2F', and so the randomisation refers to the two pathways, not the received health check. We have also corrected Table 1 to show the comparison for completing any health check by allocated pathway. For transparency, we have provided the original comparison by type of health check received to the Supplementary tables (new table S4). Note this has not notably affected the results or conclusions.

We have therefore:

- Added text summarising this distinction and justification in the methods (lines 270 & 274-77)
- Moved existing Table 1 to the Supplementary tables, and replaced with a new Table 1 reflecting the primary comparison
- checked the language throughout the results/completion rates section to be consistent with this (eg line 358-60, 364, 371-76)
- Added text to the discussion to ensure the results are interpreted in this light (lines 451-3, 462, 473-5)
- Added a comment about possible sampling bias in making a direct comparison between DHC and F2F to the limitations section (line 572-73)

Abstract section\Outcomes:
- Given that the study had access to GP records of 6000 patients, why did the design only

include appointment, medication and referral data? In other words, why were conditions, lab results, and procedures not included in this analysis?

To clarify, the researchers did not have direct access to GP records, just summaries and data provided to us by the GP Federation (who hold NHS patient data) under data sharing agreements. We were only able to access data on appointments, medications and referrals. We have clarified this in the data section lines 243-45:

> Note that due to limitations of **data sharing agreements**, these relate to any appointments made within the time frame **for any medical purpose**, and may not be directly **as a result of** a health check.

Abstract\Results:

- Because DHC is a digital intervention, why did the study excluded patients who had moved away?

NHS health check services are offered at a local authority level, and so any patient who had moved away would fall under a different local authority which would not offer DHC, or have the means to incorporate DHC outcomes into their systems.

In addition, both those invited to F2F directly and those allocated to DHC who chose to take up the F2F option instead would only be able to do so within the local authority.

- Risk of reporting bias: The results section starts with reporting on the DHC "uptake", whereas the outcomes section never mentioned the word "uptake"? To control the risk of reporting bias, the authors need to be explicit in defining/detailing the primary (and secondary if any) outcome measures, and report accordingly in the result section.

We have changed the outcome text to define uptake specifically in terms of completion of the F2FD/DHC pathways (line 27):

> The primary **outcome was uptake of any type of health check, either** a completed F2F appointment or completion of the DHC health assessment section.

- Risk of reporting bias: In the outcome section, the cost of development was included in the primary outcome, but the result section did not report on the development cost?

The outcome is the combined cost of management and operation; we have corrected this at line 30 (note that development costs are reported separately in the main text, but are not included in the numbers reported in the abstract:

> ~~Development,~~ management and operation costs were estimated for F2F and DHC pathways.

- Risk of cofounding factors: Because DHC patients had the opportunity to switch to F2F if they wanted to, it's likely that those patients who chose to stay in DHC would favor remote interactions. Could those preferences have attributed to lower on appointments and referrals? Authors should also indicate in the abstract if it was significantly, or not significantly, lower.

We have added the p-value to the abstract (line 39).

We agree that those who chose DHC may be more likely to prefer remote interactions, and so it may explain the lower follow-up appointments. We have added the following to the discussion (lines 565-67):

> It is also possible that those who prefer remote interactions may be more likely to choose DHC over F2F, and also less likely to follow up with a face-to-face appointment, which may contribute.

and similar text in the limitations (lines 579-80).

- Because the study has access to the full GP records, should the comparison consider the evaluation of appointments, medication and referral, before and after the DHC intervention.

As mentioned above, unfortunately we did not have access to full GP records, and so are unable to make this comparison.

- "Compared to DHC costs of £68.48 for health assessment only, £134.46 including the support section, and £1479.01 per user with completed physical measures". Please, refine/simplify this sentence. It is clear how much is the estimated total measurement and operation costs per user for DHC?

We have edited this sentence as follows (line 40-41):

> The estimated total management and operation costs for F2F were £154.80 per user, compared to **total management and operation costs for** DHC costs of £68.48 **per user** for health assessment only, £134.46 including the support section, and a total cost of £1479.01 per user with completed physical measures.

- Qualitative measure – it is important to learn more about the patient sentiment, in particular for those who chose to opt for F2F, and leave DHC? It will be insightful to learn if the reported lower appointments and referrals in the DHC cohort was, in part, because those DHC patients generally disengaged with in-person appointment/visits to the GP, and hence did not opt to go F2F and stay in DHC. The NIHR 2019 report (Informing NHS policy in 'digital-first primary care': a rapid evidence synthesis (nih.gov) addresses some of those concerns: "Although uptake of alternative consultation technologies in primary care was found to be low, it was unclear if this was due to GPs applying the use of alternatives selectively, patients not being aware of their availability or not wishing to use them, or problems with implementation. The authors also noted a lack of evidence relating to the roles of team members, such as reception staff, in delivering alternatives to the face-to-face consultation"

We agree that understanding patient perspectives is important, and so the study as a whole included qualitative interviews with both patients and health care professionals. Due to word limits, we have split the study into two papers, with the current manuscript summarising the

quantitative findings, and a separate paper, currently under review at the same journal, describing the qualitative findings.

Introduction

- Risk of selective reporting bias: The authors draw a reference on how "Both CVD and type 2 diabetes are strongly associated with health inequalities" – is this topic relevant/addressed by this study? Is so, (1) please indicated in the study objectives?

This is relevant background information for the development and use of NHS health checks, and thus is important context for interpreting our findings if DHC were to be rolled out more widely across England and Wales. The purpose of the NHS health check system is early detection of CVD/T2D (line 84) and in particular to reduce health inequalities in CVD/T2D (line 89). The first aim of the study includes 'to assess the extent to which the DHC is effective at engaging those groups that are under-represented in F2F' (line117), that is whether it addresses these existing inequalities.

- "...assess the extent to which the DHC is effective at engaging those groups that are under-represented in F2F". The design methods and results do not conform with this statement. Please clarify if the objective of the DHC design was based on the under-represented patient groups in F2F? If so, the inclusion criteria need to reflect that please.

The primary aim is to compare health check completion in the F2F and DHC pathways overall, so the inclusion criteria have been chosen to reflect this, and all those eligible for health checks who were still living in the area were included. Note that whether DHC engages under-represented groups is a secondary part of that. We have reworded the objective slightly to reflect this (lines 116-17):

> To **firstly** compare the uptake of DHC and F2F, and **secondly** assess the extent to which the DHC is effective at engaging those groups that are under-represented in F2F

In terms of addressing this outcome, we have chosen not to identify a specific group as 'those less likely to engage with standard health checks' as the literature is not detailed enough to define such a group. Instead, we have analysed health check completion by demographic subgroups (lines 276-80) to address whether uptake in the DHC pathway is relatively higher for some groups than for others, including those groups identified as being under-represented (line 276). We have then interpreted these results (line 368-74) within the context of under-represented groups in the discussion (lines 472-92). We also hope that some of the clarification over the primary comparison (i.e. completion of any type of health check; see earlier response for details) helps to address this.

- With respect to the 2018 rapid evidence synthesis study (Rodgers, M., et al. 2019), the findings indicated that users of digital health interventions are likely to be younger, female and have higher income and education levels. In this case, the authors should explain how they envisioned a digital health intervention, contrary to the findings of that 2018 study, will in fact benefit under-represented patients and address healthcare inequalities.

The rationale for this was based on initial findings from a pilot of the DHC tool. We have added further details of the pilot study to motivate the current study and provide more context at lines 109-11:

> Initial piloting of the DHC (which did not include a comparator group) found high engagement among those from more deprived areas and those who did not respond to a previous F2F health check offer.

Methods\Study design
- It will be important to declare why 9000 patients were selected in this sample. Was it a form of convenience sampling?

A pragmatic sample size of 9000 patients was chosen at random from the GP record database. Please see our response to the editor's feedback above for further details and the changes we have made as a result.

- What was the rationale for the uneven split between the DHC and F2F cohorts, being 2:1 (6000:3000)? If it was bound to recruitment feasibility for DHC vs F2F, why did the split not remain even 3000 each?

The larger DHC sample was to allow us to explore the DHC process in more detail, including movement through the pathway and in particular the uptake of physical measures. We have added this rationale to the methods at line 143-5.

- What is IRAS?

IRAS Is the Integrated Research Application System, which is a system for applying for the permissions and approvals for health and social care/community care research in the UK. We have added explanation to the methods at line 151-53:

> This study uses pseudonymised demographic and health data from patient records and was approved by the **Integrated Research Application System** (IRAS) **which provides permissions and approvals for health and social care/community care research in the UK**.

- "followed by an SMS reminder 2 days later (DHC) or one week later (F2F)". It seems F2F is an existing program that makes it hard to change and align with this study design. Could the authors explain why the rational behind not sending DHC reminder after one week, instead of 2 days, to align with F2F?

The reviewer is correct that F2F is the existing process that is implemented nationally, and cannot be changed directly. As such, we have now emphasised that this is a service evaluation that compares usual practice with the DHC alternative, rather than a clinical trial that compares F2F with DHC directly (see earlier response for details).

Methods\Health check details
- What is "GP surgery"? And what's the difference between 'GP Surgery' and 'GP'?

A GP (General Practitioner) is a doctor who provides general health services to their local community and refers patients to other medical services as needed. GPs are based in a local GP surgery, and all NHS patients are registered with a GP surgery in their local community which offers a range of medical services and is the first point of call for medical issues. GP surgeries are usually responsible for managing F2F NHS Health checks.

We have added brief explanatory text at line 132 for the GP and their relationship to the GP surgery:

> General Practitioner (GP; **a doctor who provides general health services to their local community through a GP surgery**)

- Figure1 "Overview of Face-to-Face (F2F) and Digital Health Check (DHC) pathways" is not readable and distorted when zoomed in. I therefore was not able to review it.

We are sorry you were not able to view the figure. We have checked the resolution and display of the figure that we uploaded, and so this appears to be a formatting issue with the journal review system.

- "Those service users whose results". What is the service the authors are referring to? Did the authors mean to say "Those users whose results..." instead?

The service here is the NHS Health Check Programme. The term 'service user' was chosen by our patient and public involvement representatives, and so we have used it throughout the manuscript. We have add the following at line 172-74 in the Patient and Public Involvement section:

> The term 'service user' was chosen by our patient and public involvement representatives as the preferred language to refer to users of either pathway, and so we have used this term throughout this paper.

- "Those users at very high risk were advised to contact their GP clinic urgently: those who were very underweight". Perhaps, the authors could rephrase for more clarity and simplicity.

We have rephrased this sentence to read (line 207-8):

> Those users **identified as** at very high risk were advised to contact their GP clinic urgently: **that is,** those who were very underweight (BMI<16), with very high blood pressure (systolic>180mmHg, diastolic>110mmHg), or with very high HbA1c (>100 mmol/mol).

Methods\Data

- "Those from the most deprived areas were defined as residing in areas form the lowest deprivation quintile". Please, indicate and cite the SDOH scale that was used to identify the "most deprived" and "lowest deprivation quintile". In table 1, there is a reference to IMD. What is IMD?. In table S1, there is a reference to index of multiple deprivation, please provide a citation and clear reference to the year and country in scope for that index.

We have used the Index of Multiple Deprivation (IMD), the measure used by official statistics in England. We have added the missing citation at line 227, updated the definition of 'most deprived' at line 232 and checked the text and tables for consistency.

- Please rephrase and elaborate on the limitation here, or perhaps add to the limitation section. "Note that due to limitations of the data, these relate to any appointments within the time frame and may not link directly to a health check". What is the limitation? One which data, which group, and what is the magnitude and impact of this limitation to the reported findings of this study?

The limitation is that appointments, medications and referrals data cannot be directly interpreted as the outcome of a health check, but may refer to appointments for other unrelated conditions during this time frame. This limitation applies to both F2F and DHC pathways and so any bias will apply to both equally. This will therefore affect absolute estimates of appointment rates etc but not the comparison between them, provided the levels of unrelated appointments are similar.

As well as clarifications in the data section(lines 243-45 – see above), we have added more detail on the limitations of these data in the limitations section (lines 576-79):

> Due to data sharing agreements, we were also unable to attribute GP appointments, referrals and medications as a direct result of health checks. However, this limitation applies equally to both F2F and DHC pathways, and so provided the levels of unrelated appointments etc are similar, the difference will be unbiased.

We have also made corresponding changes in the Strengths and Limitations overview (line 61-62).

- "The user survey contained questions on respondent opinions of the health checks, motivation to attend health checks, satisfaction with services offered post health check, and any actions taken following health check completion". It seems we had a missed opportunity to understand why 198 patients opted for F2F instead of DHC? It will be great to learn why those patients opted out for F2F?

Unfortunately, respondents to the user survey were not linked to the main health check data, and so we cannot identify which, if any, responses were from the 198 who chose F2F instead of DHC. However this topic is discussed further in the related qualitative study which is also under review at BMJ Open at this time.

Results\Table 2

- The development cost was estimated at £261,000. This is not a static cost. There will be an ongoing operational cost for hosting and technical support and maintenance. While this cost was accounted for within the context of this study, it will important to factor in the estimated cost to sustain, scale up the website for the larger number of users on a five-years basis – given that, as indicated by the authors, local Authorities are required to offer all eligible individuals an NHS Health Check every five years.

The development cost of £261,000 includes only those part of the development (eg design of the DHC, piloting) that have already been completed and so is a static cost. The operational costs presented in Table 2 under DHC health assessment include hosting, technical support and maintenance of the DHC service (see footnote 4 to Table S13). Note these ongoing operational costs are included in the potential scale-up scenario presented in Table 2.

We appreciate that this detail is easily missed in a footnote to a supplementary table, and so we have added the following to the main manuscript in the methods for the cost analysis (line 307):

> Development costs were those relating to the pre-study phases of DHC pathway evolution in Southwark (**e.g. DHC design and piloting**).

And (line 311):

> Operational costs were those associated directly with running the service and include identification and invitation to participate, and delivery of the DHC and F2F pathways, **including the cost of hosting and technical support for the digital service**.

<u>Reviewer: 2</u>

Comments to the Author:
Line 31-31 quotes are with or without spaces, it would be good to standardize, although it is only an editing error.

We are unable to find the specific issue referenced here, but we have the checked the formatting of the manuscript to be consistent throughout.

<u>Reviewer: 3</u>

Comments to the Author:
The manuscript titled "An Evaluation of the Uptake and Effectiveness of a Digital Delivery of the NHS Health Check Service: a randomised controlled study" presents an investigation into the adoption and impact of a digital approach to a well-established public health intervention. With the growing emphasis on and urgent need for the digital delivery of health services, this study is a welcome contribution to the current literature by exploring how digital delivery methods might enhance or hinder the NHS Health Check program's reach and effectiveness. The study's relevance is underscored by its potential implications for public health policy contributing to the bedrock of evidence advocating for the swift uptake of more digital solutions.

In reviewing this manuscript, some aspects require further refinement to optimally render its outcomes' significance precisely:

1. In the methods section under the subsection "Data" you mention that you "...estimated the costs using resource and cost data supplied by Southwark staff." - Regarding this more information would be needed to understand the underlying data. Who is the mentioned Southwark staff precisely, are they employees from a specific agency in Southwark? Where is the data from? What kind of data has been provided?

This refers to the staff in the Southwark Council team responsible for running the NHS Health Check programme in the local authority, and for the design and implementation of the DHC version. Thus the resource and cost data come directly from the team responsible for allocating those resource and budgeting those costs.

We have edited this sentence to be clearer (line 249-53):

> We estimated the costs **associated with each pathway** using resource and cost data supplied by **the** Southwark **Council team who designed and implemented the DHC tool** for the DHC pathway, and data from the Southwark **Council team responsible for the running of the standard F2F NHS health check programme** and relevant literature for the F2F pathway.

2. Further explanations or references regarding the "health priorities" would help better understand the potential added value to the individuals' health. What do those engulf exactly? How are they delivered concretely (text, videos, apps, gamification, etc.)?

The health priorities were linked to the areas covered by the health check, namely: to find out physical measures if these were not known, to stop smoking, to drink less alcohol, to achieve a healthy weight, to move more, to improve mental wellbeing and to improve blood pressure, cholesterol or blood sugar levels. We have listed these at line 214-16. Users were then signposted to local and online support services; for example a smoker might be directed to tools to manage cravings, a free phone consultation on quitting via the Southwark's Healthy Lifestyle Hub and/or Southwark's digital health and wellbeing coach. We have added these examples at line 218-19.

3. One of the main limitations highlighted in the study has been the different recruitment processes for the F2F and DHC pathways. As such it would be important to characterize those further. Is there an estimation of the successfully delivered SMS messages as mobile phone numbers can be outdated in the EMIS or whatever registry extracted from? The same applies to the addresses the additional letters were sent to regarding the DHC.
Further were there any differences in the SMS reminders between the F2F and DHC pathways? Providing the message text as part of the supplemental material could be helpful regarding reproducibility and future improvements.

This is an important issue and represents a limitation of the study, but unfortunately, we do not have the data that would enable us to investigate further. We have therefore expanded the limitations section on Lines 573-76 to make this limitation clear.

4. One of the main findings was the immature process of obtaining physical measures when missing for the DHC pathway. You describe that " Those users who did not provide physical measures were offered the option of finding out as one of their health priorities, and given choices and directions to obtain measures via pharmacies, leisure centres, kiosks, GP surgeries or a home finger-prick blood test kit". It will be critical to characterize the current processes better to understand the existing potential barriers for users to obtain such measures individually. Which steps have to be taken by users for each of those options (pharmacies, leisure centres, kiosks, GP surgeries, home finger-prick blood test

kit) to receive their physical measurements?

We agree that this would be useful information to know. Unfortunately we do not have the data to answer this question but we have added this as a limitation on lines 582-83.

Overall, the manuscript provides valuable insights into the potential benefits and challenges associated with introducing digital public health services. The study's findings are particularly pertinent for policymakers, healthcare providers, and researchers interested in digital health innovation and its role in preventive care. However, to strengthen the manuscript further, the aforementioned concerns should be addressed. Addressing these issues will enhance the manuscript's clarity, rigor, and overall contribution to the field. With these revisions, this study has the potential to make a meaningful contribution to accelerate the digital health strategies within the NHS and beyond.

Thank you for your supportive comments. We hope that the edits we have made have strengthened the manuscript and will thus provide a useful contribution to the evidence.

VERSION 2 - REVIEW

Reviewer **1**

Name **Al-Durra, Mustafa**

Affiliation **University of Toronto, Centre for Global eHealth Innovation, Techna Institute, University Health Network**

Date **14-Oct-2024**

COI

The authors provided adequate and pointed responses to all my review points, with material revision to the original manuscript.

Reviewer **3**

Name **Lin, Simon**

Affiliation **Symptoma GmbH, Science Department**

Date **05-Oct-2024**

COI

Overall, the manuscript and study's findings are advancing the field of digital health and its delivery on a large scale. Particularly by making a meaningful contribution to accelerate the

digital health strategies within the NHS and beyond. The methodology is robust for the purpose of a service evaluation, and the findings are important. While a few minor noted limitations remain (i.e. barriers to physical measurements, different recruitment processes, and their impact on their success), I understand that it may not be feasible for the authors to address these limitations duly due to the lack of information and/or structural limitations. Nevertheless, these limitations do not undermine the overall validity of the study's conclusions. I recommend that the authors acknowledge these limitations more explicitly in the discussion section and encourage further research to explore these issues in more depth.

Given the strengths of this study, I recommend it for publication.
